# Restless legs syndrome among third-trimester pregnant women attending ANC Clinic in public hospitals in North Wollo Zone, Ethiopia

**DOI:** 10.1371/journal.pone.0360405

**Published:** 2026-10-15

**Authors:** Esuyawkal Mislu, Abraraw Terefe, Henok Kumsa, Solomon Moges, Mulat Ayele, Fiker Chane

**Affiliations:** 1 Department of Midwifery, College of Health Science, Woldia University, Woldia, Ethiopia; 2 College of Public Health, Ph.D. Program in Global Health and Health Security, Taipei Medical University, Taipei, Taiwan; 3 School of Medicine, College of Health Science, Woldia University, Woldia, Ethiopia; University of Florida, UNITED STATES OF AMERICA

## Abstract

**Background:**

Restless leg syndrome is uncomfortable sensations in the legs particularly while sleeping or resting at night, causing an urge to move the legs. It is a debilitating illness and has an association with quality of life and maternal and fetal outcome for pregnant women. However, RLS is underdiagnosed and poorly understood by clinicians and the general public. In Ethiopia, there was limited data on restless leg syndrome and its associated factors among pregnant women.

**Objective:**

To assess the prevalence of restless legs syndrome and associated factors among third-trimester pregnant women receiving ANC care in public hospital in North Wollo Zone, Ethiopia.

**Method:**

An institution-based cross-sectional study was conducted from November, 2023 to February, 2024. Systematic random sampling method was utilized to identify study participants. Then, an interview with a structured and pretested questionnaire and data extraction from medical charts were done for 422 women. The collected data were coded and entered using EPI DATA, and bi-variable, and multivariable logistic regression analyses were done using STATA version 16. Statistical significance was declared at a p-value less than 0.05.

**Result:**

Four hundred twenty-two eligible third-trimester pregnant women (100% response rate) participated in this study, and the prevalence was identified as 11.61%. Factors such as urban residency (AOR 5.781; 95%95% CI: 1.172, 28.501), gestational age 37 weeks or more (AOR 7.789; 95% CI: 3.027, 20.039), having a history of abortion (AOR 2.381; 95% CI: 1.081, 5.246), taking iron supplementation (AOR 0.267; 95% CI: 0.094, 0.754), and alcohol drinking during pregnancy (AOR 2.289; 95% CI: 1.049, 4.995) were significantly associated with restless legs syndrome in the third trimester of pregnancy.

**Conclusion:**

One out of ten third-trimester pregnant women had RLS, and living in urban areas, advanced gestational age, history of abortion, iron supplementation, and alcohol drinking have significant associations with RLS. Therefore, it is essential to give attention to RLS during pregnancy and provide support for pregnant women to get the recommended iron supplement and refrain from alcohol.

## Introduction

Restless leg syndrome (RLS) is a syndrome that manifests itself with uncomfortable sensations in the legs particularly while sleeping or resting at night, causing an urge to move the legs. While it cannot usually be expressed thoroughly by patients, it manifests itself in thighs, legs, and feet, and even with such complaints as tingling, chilling, itching, crushing, and burning [1,2]. It is a debilitating illness that has affected people over the centuries [3].

Many disorders, such as sleep disorders,  quality of sleep, and depression symptoms, are highly correlated with RLS [4]. Having this problem decreases the quality of sleep two to three times as compared to thus woman without RLS [5,6]. Especially during the perinatal period, this problem contributes to adverse pregnancy outcomes such as maternal-fetal morbidity, mortality, maternal anxiety and depression, and also it predisposes the offspring to various anxiety disorders and learning disabilities [4,7–9].

During pregnancy RLS related complaints are common and need attention in pregnant women [3]. Especially during the third trimester (later gestational age) the prevalence is significantly higher [10]. Many people with RLS find it difficult to fall or stay asleep. Severe RLS can cause marked impairment in life quality and also result in depression [11,12].

Restless leg syndrome prevalence is found to be between 3.9% and 15% of the general population [13,14]. However, during pregnancy the prevalence is reported up to 25% [15] and worldwide the overall pooled prevalence of RLS during pregnancy is 21% [16]. The pooled prevalence of RLS in the third trimester is 22.9% and there was a report up to [16] and decreased rapidly after delivery [15,16]. Different risk factors were known for developing RLS. Some of the risk factors include female gender, pregnancy, low iron levels, lower socioeconomic status, poor health, elderly age, comorbidity with Parkinson’s disease, positive family history of RLS, and comorbidity with psychiatric disorders [12,17].

It is not only the symptoms and magnitude of RLS that matters. It is also found that RLS has association with several pregnancy complications, including pregnancy-induced hypertension [18–20], cardiovascular disease [18–21], and gestational diabetes [22,23]. Moreover, RLS has also adverse effect on neonatal outcomes, such as preterm birth [24,25], miscarriage [26] and low birth weight [20,25].

Pregnant women with some characteristics like not taking prophylactic iron supplementation [23,27], history of premenstrual syndrome [21], history of chronic diabetes mellitus [28], later gestational age (third trimester of pregnancy) [10], sleep disruption, poor quality of sleep, higher number of stressful life events, and excessive daytime sleepiness [19] have a higher prevalence of RLS than their counterparts. A woman who experienced RLS during pregnancy is also at increased risk of baby blue [21], postpartum depression [29], recurrent RLS for future pregnancy, chronic RLS and cardiovascular disease in the future [28,30].

Contrary to findings from various studies conducted globally, RLS remains underdiagnosed and poorly understood by both clinicians and the general public [3]. Furthermore, there is limited data on the prevalence of RLS and its associated factors among third-trimester pregnant women in Ethiopia and across Africa. Therefore, this study aimed to assess the prevalence of RLS and its associated factors among third-trimester pregnant women attending antenatal care at public hospitals in North Wollo Zone, Ethiopia. The findings of this study will serve as a valuable input for health sector managers in their decision-making process. For hospital administrators and health care providers, the result will enhance understanding of RLS and its associated factors, enabling them to respond appropriately.

## Methods and materials

### Study setting

An institution-based cross-sectional study design was conducted in public hospitals of the North Wollo Zone, Amhara Regional State. North Wollo is one of the twelve zones found in Amhara region. There are 6 public hospitals (one comprehensive specialized hospital, one general hospital, and four primary hospitals) found in the study area. The capital town is Woldia, which is located 521 km away from Addis Ababa (the capital city of Ethiopia) and the geographical coordinates are 11° 50’ 0“ North, 39° 41’ 0” East [31].

Based on previous six month data found from each hospital report on average Woldia Comprehensive Specialized Hospital had 640, Lalibela General Hospital had 445, Mersa Primary Hospital had 326, Wadla Primary Hospital had 315, Kobo Primary Hospital had 280, and Meket Primary Hospital had 250 pregnant women visit for antenatal care.

### Source population

All pregnant women who visited ANC clinic in public hospitals of North Wollo Zone for ANC follow-up will be the source population.

### Study population

All third-trimester pregnant women who visited ANC clinic in public hospitals of North Wollo Zone for ANC follow-up during the data collection period were the study population.

### Eligibility criteria

#### Inclusion criteria.

All third-trimester pregnant women who visited ANC clinic in public hospitals of North Wollo Zone for ANC follow-up and volunteer to participate in the study were included.

#### Exclusion criteria.

Pregnant women who can’t provide response due to ill health status were excluded from the study.

### Sample size determination and sampling technique

#### Sample size determination.

The sample size was determined by single population proportion formula using Epi-info software with the following assumptions: margin of error 5%, confidence level 95%, proportion 0.5 (no similar study), and adding 10% non-response rate. The final calculated sample size was 422.

#### Sampling technique.

Systematic sampling technique was employed after calculating the value of k. Based on previous months’ reports of each hospital, it was expected that 3384 eligible women would visit ANC clinic during the data collection period. K/n = 3384/422 = 8. Therefore, participants were taken every 8^th^ interval. 120 from WCSH, 83 from Lalibela General Hospital, 61 from Mersa primary Hospital, 47 from Meket Primary Hospital, 59 from Wadla Primary Hospital, and 52 from Kobo Primary Hospital.

### Data collection procedure

Data were collected from November 7, 2023 to February 7, 2024 using pre-tested interviewer administered questionnaire which includes: socio-demographic characteristics of the study participants, substance use, mental and psychosocial related question, standardized restless leg syndrome related questions and medical record review checklist (S1 File.). Data collectors were six BSc Midwives after they took training; and two supervisors were assigned to monitor and take measure for any deviation from the pre designed method of data collection process accordingly. The data collection took three months. Before the actual data collection, pretest was done on 21 eligible women at Hayike Primary Hospital.

### Operational definitions

**Restless leg syndrome:** a woman was considered as she has experienced restless leg syndrome when she full fill the diagnosis criteria based on restless leg syndrome study group criteria [32].

**Good sleep quality:** a woman was categorized as she had good quality of sleep if she had PSQI score of <5 based on self-reported questionnaire [33].

**Poor sleep quality:** a woman was categorized as she had poor quality of sleep if she had PSQI score >5 based on self-reported questionnaire [33].

**Gestational age:** a woman’s gestational age was taken after calculating with the more reliable method based on the available data for individual woman using history, Ultrasound, or physical examination.

### Data quality assurance

Data quality was assured by developing structured questionnaire in English based on objectives to be addressed after reviewing of relevant literatures and it was reviewed by principal and co-investigators. The translation of questionnaire from English to Amharic was done by language expert. The data collection was done by trained data collectors and supervisors (BSc Midwives), after pretest was conducted on 5% of calculated sample size at Hayik Primary Hospital.

Any ambiguous and unsuitable questions was modified after checking of questionnaire completeness with pretest. The criteria for selecting the data collectors and supervisors were; those who have BSc Midwifery, and willing to face difficulties that may arise during the process of data collection. The data collectors and supervisors took training before the actual data collection. Additionally, the collected data were checked by supervisors for its completeness; and name of data collectors recorded so as to enhance responsibility to any incomplete data.

### Data processing and analysis

The collected data were coded and entered in to Epi data and exported to Stata version 16 for analysis. The data were checked for consistencies and missing value before analysis. Descriptive statistics like mean, standard deviation, frequencies and percentages and texts were used. Moreover, bi-variable and multivariable logistics regression analyses were done to identify independent variables association with the dependent variable. Variables with P-value <0.25 on bi-variable analysis were adjusted to identify independent associated variables. In the multivariable analysis variables having p-value <0.05 were taken as significant predictors of restless leg syndrome in the third trimester of pregnancy. Crude and adjusted odd ratios with their 95% confidence level were computed to show the association between dependent and independent variables. Then, the finding of the study was presented using text, tables and figures accordingly.

### Ethics approval and consent to participate

Approval to carry out this study was received from Ethical review board of Woldia University (Ref.No: WDU/IRB001/94). Additionally, informed written consent was obtained from each study participants prior to enrollment. The study was conducted from November 7, 2023 to February 7, 2024. Confidentiality was also assured throughout the study.

## Result

### Sociodemographic characteristics of study participants

A total of 422 third trimester pregnant women were included in the study with a response rate of 100%. Of these 177 (41.94%) were on the age between 25–29 years, 127(30.09%) were primary school and 326(77.25%) were urban dwellers (Table 1).

**Table 1:** Sociodemographic characteristics of study participants (n = 422).

Variables	Categories	Frequency	Percent
Age	≤19	35	8.29
	20-24	87	20.62
	25-29	127	30.09
	30-34	106	25.12
	≥35	67	15.88
Residence	Urban	326	77.25
	Rural	96	22.75
Educational level	No formal education	83	19.67
	Primary School	127	30.09
	Secondary School	115	27.25
	College and above	97	22.99
Marital Status	Married	389	92.18
	Not married	33	7.82
Occupation	Housewife	261	61.85
	Government employee	61	14.45
	Self-employee	46	10.90
	Farmer	34	8.06
	Other	20	4.74

### Medical and obstetric characteristics of participant women

One hundred sixty-two (38.39%) of the participants were nulli-parous and most of the participants (89.81%) pregnancy were planned. Additionally, 6.87% of the participants had history of chronic disease and 16.11% had anemia (Table 2).

**Table 2:** Medical and obstetric characteristics of study participants (n = 422).

Variables	Categories	Frequency	Percent
Parity	Nulli-para	162	38.39
	Primi-para	109	25.83
	Multi-para	151	35.78
Type of pregnancy	Planned	379	89.81
	Not planned	43	10.19
Gestational age in weeks	28-33 weeks	225	53.32
	34-36 weeks	107	25.35
	≥37weeks	90	21.33
History of abortion	Yes	92	21.85
	No	329	78.15
History of chronic disease	Yes	29	6.87
	No	393	93.13
Anemia	Yes	68	16.11
	No	354	83.89
Iron supplementation	Yes	388	91.94
	No	34	8.06

### Personal habit and mental health related characteristics of study participant

Sixteen (3.79%) women were chewed chat during the current pregnancy and 86(20.38%) of the participants had antenatal depression (Table 3).

**Table 3:** Personal habits and mental health related characteristics of study participants.

Variables	Categories	Frequency	Percent
Chewing chat during the current pregnancy	Yes	16	3.79
	No	406	96.21
Alcohol drinking during pregnancy	Yes	165	39.1
	No	257	60.9
Antenatal depression	Yes	86	20.38
	No	336	79.62
Sleep quality	Good	171	40.52
	Poor	251	59.48
Antenatal Physical Exercise	Yes	123	29.15
	No	299	70.85

### Prevalence of Restless Legs Syndrome among third trimester pregnant women

This study identified that 49 women from the 422 participants fulfill the criteria for restless legs syndrome making the prevalence as 11.61% (95%CI: 8.87%, 15.04%). Additionally the restless legs syndrome rating scale showed that 0.24%, 6.4%, 4.5%, and 0.47% of the respondents had mild, moderate, sever, and very sever type of restless legs syndrome.

### Factors associated with Restless Legs Syndrome among third trimester pregnant women

On the bi-variable analysis eleven variables: residence, educational level, occupational status, gestational age, history of abortion, taking iron supplementation, chat chewing, alcohol drinking during pregnancy, antenatal exercise, depression, and quality of sleep had a p-value less than 0.25. These variables were entered to multivariable logistic regression analysis which results in urban residency AOR 5.781(1.172, 28.501), gestational age 37 weeks or more AOR 7.789(3.027, 20.039) as compared to 28–32 weeks of gestation, having a history of abortion AOR 2.381(1.081, 5.246), taking iron supplementation AOR 0.267(0.094, 0.754), and alcohol drinking during pregnancy AOR 2.289(1.049, 4.995) were significantly associated with restless legs syndrome during the third trimester of pregnancy (Table 4).

**Table 4:** Bi-variable and multivariable logistic regression result for RLS among pregnant women in the study area (n = 422).

Variable/ Categories	RLS	No RLS	COR(95%CI)	AOR(95%CI)	p-value
**Residence**					
Rural	7	89	Ref.	Ref.	
Urban	42	284	1.880(0.815, 4.332)	5.781(1.172, 28.501)	**0.031**
**Educational Level**					
No formal Education	8	75	Ref.	Ref.	
Primary School	21	106	1.857(0.780,4.417)	1.543(0.8231, 21.481)	0.185
Secondary School	8	107	0.700(0.251, 1.950)	1.516(0.333, 6.890)	0.590
College and above	12	85	1.323(0.513, 3.411)	4.079(0.750, 22.175)	0.104
**Occupational Status**					
Housewife	24	237	Ref.	Ref.	
government employee	6	55	1.077(0.420, 2.761)	0.509(0.134, 1.933)	0.322
Self-employee	8	38	2.078(0.870, 4.964)	1.381(0.484, 3.942)	0.546
Farmer	7	27	2.560(1.008, 6.497)	3.358(0.745, 31.689)	0.102
Other	4	16	2.468(0.7636, 7.980)	1.353(0.360, 5.084)	0.654
**Gestational age**					
28-34 weeks	10	215	Ref.	Ref.	
34-37 weeks	14	93	3.236(1.387, 7.551))	2.464(0.950, 6.393)	0.064
≥37 weeks	25	65	8.269(3.308, 18.112)	7.789(3.027, 20.039)	**0.000**
**History of Abortion**					
Yes	20	72	2.883(1.543, 5.386)	2.381(1.081, 5.246)	**0.031**
No	29	301	Ref.	Ref.	
**Taking Iron tablet**					
Yes	40	348	0.319(0.139, 0.731)	0.267(0.094, 0.754)	**0.013**
No	9	25	Ref.	Ref.	
**Chat chewing during pregnancy**					
Yes	4	12	2.674(0.827, 8.643)	3.241(0.778, 13.505)	0.106
No	45	361	Ref.	Ref.	
**Alcohol Drinking during pregnancy**					
Yes	28	137	2.296(1.255, 4.200)	2.289(1.049, 4.995)	**0.037**
No	21	236	Ref.	Ref.	
**Antenatal Physical Exercise**					
Yes	22	101	2.194(1.195, 4.028)	2.049(0.840, 4.994)	0.115
No	27	272	Ref.	Ref.	
**Antenatal Depression**					
Yes	15	71	1.876(0.969, 3.631)	1.877(0.651, 5.415)	0.244
No	34	302	Ref.	Ref.	
**Quality of Sleep**					
Poor	30	141	2.597(1.409, 4.788)	1.711(0.758, 3.862)	0.196
Good	19	232	Ref	Ref.	

## Discussion

Restless Legs Syndrome is linked to different conditions like iron deficiency, chronic illnesses, quality of life, pregnancy and pregnancy outcomes. This institution-based cross-sectional study aimed to determine the prevalence and associated factors of RLS during the third trimester of pregnancy among women attending antenatal care. The findings revealed a prevalence of 11.61% (95% CI: 8.87%, 15.04%). This prevalence aligns with studies conducted in Taiwan (10.4%) [34], Pakistan (11%) [35], and China (16.1%) [36]. However, this prevalence is lower than the 22% prevalence of RLS in the meta-analysis of third trimester pregnant women [37], 19% in University hospital of Aachen, Germany [23], 24.1% in King Abdulaziz Medical City in Riyadh, Saudi Arabia [38], 64.4% in Turkey [39], 50.3% in Brazil [40], 31.2% in New York [41], and the 22% prevalence of RLS in the meta-analysis of third trimester pregnant women [37].

The discrepancy in the prevalence of RLS in these studies might be related to population characteristics such as genetics and ethnic diversity [42,43], as well as the higher prevalence of obesity in high- and middle-income countries [44]. Access to health care and well-developed prenatal care systems may influence symptom reporting. In developed countries such as Germany, and Britain, higher awareness and diagnosis rates due to advanced healthcare systems may lead to increased symptom reporting, which can significantly affect the observed prevalence [45].

The multivariable logistic regression analysis identified five statistically significant factors associated with RLS. Women residing in urban areas were 5.781 times more likely to have RLS compared to those living in rural areas. This finding is consistent with a study conducted in India [46] and can be explained by several factors. Urban environments are often associated with more sedentary lifestyles, which reduce physical activity, a known protective factor against RLS [47]. Additionally, environmental stressors such as noise, pollution, and the fast pace of urban life can contribute to higher stress levels and poor sleep quality, both of which are linked to RLS [48]. Furthermore, women in urban areas may have better access to healthcare and greater awareness of health issues, leading to more frequent diagnosis and reporting of RLS symptoms. This suggests the need to promote healthy lifestyles in urban populations and to improve access to healthcare services in rural areas.

Women at 37 weeks of gestation or more were found to be 7.789 times more likely to experience RLS compared to those at 28–32 weeks. This strong association can be attributed to several physiological and psychological changes that occur during late pregnancy. As pregnancy progresses, increased body weight and the growing fetus place additional pressure on the lower extremities, compressing nerves and restricting blood flow to the legs, both known triggers for RLS. Hormonal fluctuations in the late third trimester and influence dopaminergic pathways in the brain, disrupting movement regulation and sensory perception, thereby exacerbating RLS symptoms [49]. Additionally, reduced mobility and physical activity due to discomfort and fatigue limit the natural relief for RLS [50]. Psychological factors such as stress and anxiety about impending labor and delivery may also intensify symptoms [48,50]. Together, these factors highlight the importance of close monitoring and supportive interventions to help alleviate RLS symptoms and improve the quality of life for women nearing the end of pregnancy.

A history of abortion was found to be significantly associated with an increased likelihood of developing RLS, with affected women being 2.381 times more likely to report the condition. This association may be linked to both psychological and physiological factors [35]. Abortion, whether spontaneous or induced, can be a source of significant emotional stress and trauma, potentially leading to chronic stress or anxiety, both known to be associated with RLS [48]. Additionally, repeated pregnancies or pregnancy losses may deplete iron stores and other essential nutrients, increasing the risk of RLS in subsequent pregnancies [51]. This finding highlights the importance of comprehensive antenatal care, including psychological support and nutritional monitoring, particularly for women with a history of abortion.

Iron supplementation was found to be protective against RLS, with women taking iron supplements being 73.3% less likely to develop the condition. This finding supports the well-established link between iron deficiency and RLS, as iron is crucial for the synthesis of dopamine, a neurotransmitter that regulates movement [23,34,52]. During pregnancy, iron demands increase significantly to support fetal development, and inadequate iron levels can impair dopamine production, contributing to RLS symptoms [53]. Iron supplementation helps restore iron levels, enhance dopamine function, and alleviate RLS symptoms [49,51]. This protective effect highlights the importance of monitoring iron status and ensuring adequate intake through diet or supplements to improve maternal health and reduce RLS-related discomfort during pregnancy.

Alcohol consumption during pregnancy was found to be a significant predictor of RLS, with women who consumed alcohol being 2.289 times more likely to experience the condition. This association highlights the impact of modifiable lifestyle factors on RLS. Alcohol disrupts several neurological pathways, particularly those involved in movement and muscle control [54]. It affects the central nervous system, including the dopaminergic pathways, which play a key role in regulating leg movement and are closely linked to RLS [17,55]. Additionally, alcohol consumption can contribute to nutritional deficiencies, such as a lack of essential vitamins and minerals, including iron [56]. These factors suggest that alcohol intake during pregnancy can exacerbate RLS symptoms and highlight the importance of avoiding alcohol to reduce RLS risk and promote both maternal and fetal health.

Despite the valuable insights provided by this study, certain limitations should be acknowledged. The cross-sectional nature of the study limits the ability to establish causal relationship between the identified factors and RLS. Additionally, the reliance on self-reported data particularly on lifestyle factors may introduce recall bias which could have influenced the findings.

## Conclusion and recommendations

This study found that approximately one in ten pregnant women in their third trimester experienced RLS. Several factors were significantly associated with RLS including urban residence, late gestational age, primary education level, history of abortion, iron supplementation, and alcohol consumption during pregnancy. Women in late pregnancy (37 weeks or more), those with a history of abortion, and those living in urban areas were at higher risk, likely due to a combination of hormonal changes, stress, and limited access to health resources. In contrast, iron supplementation appeared to be protective, underscoring the importance of adequate iron intake in preventing or alleviating RLS symptoms. Alcohol consumption emerged as a modifiable risk factor that may exacerbate RLS, emphasizing the need for lifestyle adjustments during pregnancy.

Based on these findings, several recommendations are proposed for key stakeholders. Pregnant women and their families should ensure adherence to iron supplementation, avoid alcohol consumption, and maintain a balanced, nutritious diet to minimize the risk of RLS. Healthcare providers should screen for RLS symptoms, especially in the third trimester, ensure iron supplementation, and offer psychological support when necessary. Public health authorities are encouraged to promote education on the importance of iron intake and the risks associated with alcohol consumption during pregnancy, while also working to improve equitable access to prenatal care. Community organizations, including NGOs, can play a vital role in supporting awareness efforts and distributing educational resources. Lastly, future research should consider diverse populations and adopt longitudinal study design to better understand the development, progression, and outcomes of RLS during and after pregnancy.

## Supporting information

S1 File English and Amharic Version Questionnaire with its information sheet and consent form. (DOCX)

## Abbreviations

ANC: Antenatal care
CI: Confidence interval
PSQI: Pittsburgh sleep quality index
RLS: Restless legs syndrome
