# Supplementary material for: Restless legs syndrome among third-trimester pregnant women attending ANC Clinic in public hospitals in North Wollo Zone, Ethiopia
Source: PLoS One. 2026 Oct 15;21(10):e0360405. doi: 10.1371/journal.pone.0360405 (PMC13671541; doi:10.1371/journal.pone.0360405)
Supplement: Supplementary file 1 [file pone.0360405.s001.docx]

# Annexes

## Information Sheet

You have been chosen to participate in this study entitled “Restless legs syndrome and associated factors among third trimester pregnant women attending ANC clinic in North Wollo Zone public hospitals”. Please listen me the following statements, and if you have any unclear questions you can ask before you agree to participate.

1. **Topic**: Restless legs syndrome and associated factors among third trimester pregnant women attending ANC clinic in North Wollo Zone public hospitals, Ethiopia 2023/4.

2. **Objective of the study**: The objective of this study is to assess the prevalence of Restless legs syndrome and associated factors among third trimester pregnant women in the study area. The information you are going to provide will be helpful for health care providers, higher officials, the community, government and future pregnant women to understand this health problem and design a strategy to manage restless legs syndrome and promote positive experience of pregnant women.

3. **Participation Procedure and guidelines**; the information you provide will be kept completely anonymous. Your name will not be written on the form and your answers are completely confidential. The questions are prepared in English, and Amharic language as a structured interviewer administered questionnaire. It will take about 20-25 minutes to complete the interview.

4. **Participant benefit and risk**; your participation in the study does not involve risks. You might feel some mild discomfort from responding to some items on the questionnaires, but the risk of discomfort is not greater than other normal activities you have in the home. You also may experience some benefits from participating in this study.

5. **Right to refuse or withdraw**: You do not have to answer any question that you do not want to answer. You can end the participation in the study at any time you want, without losing any of your right.

6. **Persons to Contact**: this research project was reviewed and approved by the Ethical clearance committee of the Woldia University. If in case you want to know more information about the research and its undertakings, you can contact the committee through the address of the principal investigator below.

Esuyawkal Mislu (MSc in Midwifery). Email: [esuyawkalmislu@gmail.com](mailto:esuyawkalmislu@gmail.com)

Phone number: +251919453257

## Informed consent form

Good morning/ afternoon. My name is ________________. I am working as a data collector in a research conducted on restless leg syndrome among pregnant women attended ANC clinic in public hospitals of north wollo zone. You are invited to participate in a research conducted by Woldia University academic staffs. Your participation is voluntarily. We would like to reassure you that your participation on this research will not affect any of your care or benefit that you are getting from this hospital. We are asking you to take part in the study because we are trying to explore more about referral delay for emergency obstetric care and its associated factors. Please take as much time as you need to read/listen the information sheet. If you are willing to participate in this study, you need to understand and give your consent. Then, you will be requested to give your response. This study includes both questionnaire and medical chart review. All of your response will be kept confidential and it will take approximately 30-40 minutes.

Are you voluntary to participate in this study? (If yes) your signature ____________

Name of data collector__________________________ Date_____ Signature ____________

## English Version Questionnaire

# I) Socio demographic characteristics of study participants

| **S/N** | **Questions** | **Response** |
| --- | --- | --- |
| 101 | Age | ____________ (in completed years) |
| 102 | Residence | 1. Urban  2. Rural |
| 103 | Educational status | 1. No formal education  2. Elementary  3. High school (grade 9-12)  4. College and above |
| 104 | Marital status | 1. Single  2. married  3.divorced  4.widowed |
| 105 | Occupation | 1. House wife  2. Government employee  3. Private employee  4. Self-employee  5. Farmer  6. Student  7. Daily laborers  8. Others specify------------------ |
| 106 | Average Monthly Income | _______________________ Ethiopian birr |

# II) Pregnancy and medical history

| **S/N** | **Questions** | **Response** | | |
| --- | --- | --- | --- | --- |
| 201 | Gravidity/ parity | Gravida _______  Para ____ | | |
| 202 | Gestational age (more reliable) | ______ Weeks | | |
| 203 | Is the current pregnancy planned? | 1. Yes 2. No | | |
| 204 | Have abortion history? | 1.Yes 2.No | | |
| 205 | Presence of co-morbid HTN/ DM | 1. Yes 2. No | | |
| 206 | Presence of any complication during current pregnancy? (E.g., APH pre-eclampsia/eclampsia/ gestational HTN, PROM, infection) | 1. Yes (specify) ______________  2. No | | |
| 207 | Hemoglobin level | _____________ | | |
| 208 | BP(convenient arm) | | Systolic _______ mmHg  Diastolic _____ mmHg | |
| 209 | Iron and foliate supplementation | | | 1. Yes 2. No |
| 210 | For how many months do you took iron supplementation (in month) | | | ________ month |

# III) Substance use assessment (alcohol intake and chat chewing)

| **S/N** | **Questions** | **Response** |
| --- | --- | --- |
| 301 | Have you ever chewed chat? | 1. Yes 2. No  **If No🡪 skip to Q 303** |
| 302 | Have you chewed chat during this pregnancy? | 1. Yes 2. No |
| 303 | Have you ever drunk alcohol? | 1. Yes 2. No  **If No🡪skip to Q 306** |
| 304 | Have you drink alcohol during this pregnancy? | 1. Yes 2. No  **If No🡪skip to Q 306** |
| 305 | What type of alcohol do you drink? Specify (e.g. Beer, Tela, Teje, Areqe, wine, …) | ________, _____________, _________________, ___________ |
| 306 | Do you drink caffeinated drinks (e.g. tea, coffee, cola)? Please describe how many per day | 1. Yes _________ (Specify) 2. No |

# IV) Sleep hygiene Index

| S/N | **Sleep hygiene Index Questions** | **Response** | | | | |
| --- | --- | --- | --- | --- | --- | --- |
|  |  | Always | Frequently | Sometimes | Rarely | Not at all |
| 401. | I sleep on an uncomfortable bed (for example: poor mattress or pillow, too much or not enough blankets). |  |  |  |  |  |
| 402. | I sleep in an uncomfortable bedroom (for example: too bright, too stuffy, too hot, too cold, or too noisy). |  |  |  |  |  |
| 403. | I do important work before bedtime (for example: preparing food for the next day, schedule, or study). |  |  |  |  |  |
| 404. | I do something that may wake me up before bedtime (for example: cleaning home, phone calling, use the internet, or video game). |  |  |  |  |  |
| 405. | I use alcohol, tobacco, or caffeine within 4 hours of going to bed or after going to bed. |  |  |  |  |  |
| 406. | I think, plan, or worry when I am in bed. |  |  |  |  |  |
| 407. | I use my bed for things other than sleeping (for example, watch television, read, eat, or study). |  |  |  |  |  |
| 408. | I exercise to the point of sweating within 1 hour of going to bed. |  |  |  |  |  |
| 409. | I get out of bed at different times from day to day. |  |  |  |  |  |
| 410. | I go to bed at different times from day to day. |  |  |  |  |  |
| 411. | I stay in bed longer than I should two or three times a week. |  |  |  |  |  |
| 412. | I take daytime naps lasting two or more hours. |  |  |  |  |  |
| 413. | I go to bed feeling stressed, angry, upset, or nervous. |  |  |  |  |  |
| 414. | Which position of sleep did you prefer(most of the time) to go to sleep | A. Supine  B. Left lateral  C. Right lateral  D. Prone  E. Other, ________________ | | | | |

# V) Standardized Sleep quality measurement (PSQI) questions

| **Instructions**: The following questions relate to your usual sleep habits during the past month only. Your answers should indicate the most accurate reply for the majority of days and nights in the past month. | | | | | |
| --- | --- | --- | --- | --- | --- |
| During the past month,  1. When have you usually gone to bed? ___________________  2. How long (in minutes) has it taken you to fall asleep each night? _______________  3. When have you usually gotten up in the morning? ___________________  4. A. How many hours of actual sleep do you get at night? (This may be different than the number of hours you spend in bed) _____________  B. How many hours spend in bed per night? __________ | | | | | |
| 5. During the past month, how often have you had trouble Sleeping because you… | | Not during the last month(0) | Less than once a week(1) | Once or twice a week (2) | Three or  more  times a week(3) |
| a. Cannot get to sleep within 30 minutes | |  |  |  |  |
| b. Wake up in the middle of the night or early morning | |  |  |  |  |
| c. Have to get up to use the bathroom | |  |  |  |  |
| d. Cannot breathe comfortably | |  |  |  |  |
| e. Cough or snore loudly | |  |  |  |  |
| f. Feel too cold | |  |  |  |  |
| g. Feel too hot | |  |  |  |  |
| h. Have bad dreams | |  |  |  |  |
| i. Have pain | |  |  |  |  |
| j. Other reason(s), please describe, including how often you have had trouble sleeping because of this reason(s): | |  |  |  |  |
| 6. During the past month, how often have you taken medicine (prescribed or “over the counter”) to help you sleep? | |  |  |  |  |
| 7. During the past month, how often have you had trouble staying awake while traveling, eating meals, or engaging in social activity? | |  |  |  |  |
| 8. During the past month, how much of a problem has it been for you to keep up enthusiasm to get things done? | |  |  |  |  |
| 9. During the past month, how would you rate your sleep quality overall? | | Very  good (0) | Fairly  good(1) | Fairly bad(2) | Very bad(3) |
| 10. Do you take naps during the day? | 1. Yes If YES, for how long? __________ min  2. No | | | | |

# VI) Questions related to Restless Leg Syndrome

| **S/N** | **Questions** | | | **Answer** |
| --- | --- | --- | --- | --- |
| 501 | Desire to move the extremities usually associated with discomfort or disagreeable sensations in the extremities. | | | 1. Yes 2. No  If No🡪 Skip to the net part |
| 502 | Motor Restlessness-patients move to relieve the discomfort, for example walking, or to provide a counter-stimulus to relieve the discomfort, for example, rubbing the legs. | | | 1. Yes 2. No |
| 503 | Symptoms are worse at rest with at least temporary relief by activity. | | | 1. Yes 2. No |
| 504 | Did this symptom worsening of symptoms later in the day or at night? | | | 1. Yes 2. No |
| Exceptions—If the participant previously met IRLSSG criteria and took medication with subsequent significant alteration of symptoms.  Exception—The patient at one time got relief of symptoms by activity but is now so severe that no such relief is possible.  Exception—The patient at one time was worse later in the day or at night, but is now so severe that symptoms are equal day and night. | | | | |
| 505 | Do you have similar problem in the past pregnancy? | | 1. Yes 2. No 3. It is my first pregnancy | |
| 506 | Do you have a family history of this problem? | 1. Yes 2. No 3. I don’t know | | |

# VII) Restless Legs Syndrome Rating Scale (this is for those women with restless legs syndrome)

| S. No. | Restless Legs Syndrome Rating Scale Questions | (4) Very severe | (3) Severe | (2) Moderate | (1) Mild | (0) None |
| --- | --- | --- | --- | --- | --- | --- |
| 601 | Overall, how would you rate the RLS discomfort in your legs or arms? |  |  |  |  |  |
| 602 | Overall, how would you rate the need to move around because of your RLS symptoms? |  |  |  |  |  |
| 603 | Overall, how much relief of your RLS arm or leg discomfort do you get from moving around? | (4) No relief | (3) Slight relief | (2) Moderate relief | (1) almost complete relief | (0) **No RLS symptoms** |
| 604 | Overall, how severe is your sleep disturbance from your RLS symptoms? |  |  |  |  |  |
| 605 | How severe is your tiredness or sleepiness from your RLS symptoms? |  |  |  |  |  |
| 606 | Overall, how severe is your RLS as a whole? |  |  |  |  |  |
| 607 | How often do you get RLS symptoms? | 6-7 days a wk | 4-5 days a wk | 2-3 days a wk | ≤1 day a wk | None |
| 608 | When you have RLS symptoms, how severe are they on an average day? | ≥8 hrs/ day | 3-8 hrs/day | 1-3 hrs/day | <1hr/day | None |
| 609 | Overall, how severe is the impact of your RLS symptoms on your ability to carry out your daily affairs, for example carrying out a satisfactory family, home, social, school, or work life? |  |  |  |  |  |
| 610 | How severe is your mood disturbance from your RLS symptoms–for example angry, depressed, sad, anxious, or irritable? |  |  |  |  |  |
| How would you rate your symptom in the past week?  1 2 3 4 5 6 7 8  MILD MODERATE SEVERE VERY SEVERE | | | | | | |

# VIII) Edinburgh Antenatal Depression Scale

| S. No. | Edinburgh Antenatal Depression Scale  Questions  **In the past 7 days**: | As much as I always could | Not quite so much now | Definitely not so much now | Not at all |
| --- | --- | --- | --- | --- | --- |
| 701 | I have been able to laugh and see the funny side of things |  |  |  |  |
| 702 | I have looked forward with enjoyment to things |  |  |  |  |
| 703 | I have blamed myself unnecessarily when things went wrong |  |  |  |  |
| 704 | I have been anxious or worried for no good reason |  |  |  |  |
| 705 | I have felt scared or panicky for no very good reason |  |  |  |  |
| 706 | Things have been getting on top of me |  |  |  |  |
| 707 | I have been so unhappy that I have had difficulty sleeping |  |  |  |  |
| 708 | I have felt sad or miserable |  |  |  |  |
| 709 | I have been so unhappy that I have been crying |  |  |  |  |
| 710 | The thought of harming myself has occurred to me |  |  |  |  |

# IX) Physical exercise during pregnancy related questions

| **S/N** | **Question** | **Answer** | | |
| --- | --- | --- | --- | --- |
| 801 | Did you advised to minimize physical exercise during pregnancy by health care provider for having medical indication? | 1. Yes 2. No | | |
| 802 | Did you practice exercise before current pregnancy | 1. Yes 2. No | | |
| 803 | Did you practice exercise during current pregnancy? | 1. Yes 2. No | | |
|  | | No at all | Yes daily for <30 mints or weekly for < 3 days/ <150 mints | Yes daily ≥30 mints or weekly for ≥3 days/ ≥150mints |
| 804 | Did you have practiced walking exercise? |  |  |  |
| 805 | Did you have practiced dancing exercise? |  |  |  |
| 806 | Did you have practiced cycle driving exercise? |  |  |  |
| 807 | Did you have practiced breathing exercise? |  |  |  |
| 808 | Did you have practiced pelvic floor exercises? |  |  |  |
| 809 | Did you have practiced ankle and toe exercise? |  |  |  |

**-------Thank You------**

# ክፍል 1፡ የመረጃ መስጫ ቅጽ

እርስዎ “በሰሜን ወሎ ዞን በሚገኙ የመንግስት ሆስፒታሎች ለእርግዝና ክትትል ሰባት ወር (28 ሳምንት) እና ከዚያ በላይ ሆኗቸው በሚመጡ ነፍሰ ጡር እናቶች ላይ የሬስትለስ ሌግስ ሲንድሮም (Restless legs syndrome) ስርጭት እና ተያያዥ ምክንያቶች በሚጠናበት ምርምር ላይ የጥናቱ አካል ሆነው እንዲሳተፉ ተመርጠዋል። እባክዎ የሚከተሉትን መግለጫዎች ተከታትለው ግልጽ ያልሆኑ ጥያቄዎች ካሉዎት ለመሳተፍ ከመስማማትዎ በፊት መጠየቅ ይችላሉ።

1. **የጥናቱ ርዕስ፡-** “በ 2016 ዓ/ም በሰሜን ወሎ ዞን በሚገኙ የመንግስት ሆስፒታሎች የሬስትለስ ላግስ ሲንድሮም (Restless legs syndrome) ስርጭት እና ተያያዥ ምክንያቶች ሰባት ወር (28 ሳምንት) እና ከዚያ በላይ ሆኗቸው ለእርግዝና ክትትል በሚመጡ ነፍሰ ጡር እናቶች ላይ።” *‘Re stless legs syndrome and associated factors among third trimester pregnant women attending ANC clinic in North Wollo Zone public hospitals’*
2. **የጥናቱ ዓላማ፡-** የዚህ ጥናት ዓላማ በጥናቱ አካባቢ ከሰባት ወር (28 ሳምንት) የሆናቸው ነፍሰ ጡር እናቶች ላይ የሬስትለስ ላግስ ሲንድሮም ስርጭትን እና ተያያዥ ምክንያቶችን ለመገምገም ነው። የምትሰጡን መረጃ ለጤና አጠባበቅ ሰራተኞች፣ ከፍተኛ ባለሥልጣናት፣ ህብረተሰብ፣ መንግስት እና ወደፊት ልጅ የመውለድ እቅድ ላላቸው እናቶች ይህንን የጤና ችግር ተረድተው ለመቆጣጠር እና የነፍሰ ጡር ሴቶችን አወንታዊ ልምምዶችን ለማሳደግ የሚረዳ ነው።
3. **የተሳትፎ አካሄድ እና መመሪያዎች፦** እርስዎ በሚሰጡት መረጃዎች ላይ በሙሉ ስምዎ የማይገለጽ በመሆኑ መልሶችዎ ሙሉ በሙሉ ሚስጥራዊ ናቸው።መጠየቁ በእንግሊዝኛና በአማርኛ ቋንቋዎች የተዋቀረ ሲሆን ቃለ መጠይቅ አድራጊው መጠይቁን የሚመራው ይሆናል። ይህንን ቃለ መጠይቅ ለማጠናቀቅ ከ20-25 ደቂቃ ይወስዳል።
4. **የተሳታፊዎች ጥቅማጥቅም እና ተያያዥ አደጋ**፦ በጥናቱ በመሳተፎ ምንም አይነት አደጋ አያጋጥምዎትም። በመጠይቁ ላይ ላሉት አንዳንድ ጥያቄዎች ምላሽ ሲሰጡ መጠነኛ የሆነ ምቾት ማጣት ሊሰማዎት ይችላል፣ ነገር ግን በቤት ውስጥ ካሉት ሌሎች የተለመዱ ተግባራት አይበልጥም። እንዲሁም በዚህ ጥናት ውስጥ በመሳተፍም የተለየ የገንዘብ ወይም በአይነት የሚገኝ ጥቅማጥቅም የለም፥ አንዳንድ ጥቅሞችን ግን ሊያገኙ ይችላሉ።
5. **ያለመሳተፍ ወይም ጥናቱን የማቋረጥ መብት፡** እርስዎ መመለስ ለማትፈልጉት ማንኛውም ጥያቄ መመለስ አይጠበቅብዎትም። ማንኛውንም መብትዎን የሚነካ ጉዳይ ካጋጠመዎት በማንኛውም ጊዜ የጥናቱን ተሳትፎ ማቆም/ማቋረጥ ይችላሉ።
6. **ሊገናኙዋቸው የሚገቡ ሰዎች፡-** ይህ የምርምር ፕሮጀክት በወልድያ ዩኒቨርሲቲ የሥነ ምግባር ኮሚቴ ታይቶ ጸድቋል። ስለ ጥናቱ የበለጠ መረጃ ማወቅ ከፈለጉ፣ ከዚህ በታች ባለው ዋና ተመራማሪ አድራሻ በኩል ኮሚቴውን ማነጋገር ይችላሉ።

መ/ር እሱያውቃል ምስሉ (ወልድያ ዩኒቨርሲቲ)

ኢሜይል፡ esuyawkalmislu@gmail.com

ስልክ ቁጥር፡ +251919453257

**በመረጃ የተደገፈ የስምምነት መግለጫ ቅጽ**

ጤና ይስጥልን። የኔ ስም ________________ሲሆን በሰሜን ወሎ ዞን በሕዝብ ሆስፒታሎች የቅድመ ወሊድ ክትትል በሚያደርጉ ነፍሰ ጡር እናቶች ላይ የሚከሰት የ እግር በሽታዎች ሲንድሮም ላይ በተደረገ ጥናት መረጃ ሰብሳቢ ሆኜ እየሰራሁ ነው።እርስዎ በወልድያ ዩኒቨርሲቲ የአካዳሚክ ባለሙያዎች በሚካሄደው ጥናት ላይ እንዲሳተፉ ተጋብዘዋል። የእርስዎ ተሳትፎ በፈቃደኝነት ላይ የተመሰረተ ሲሆን በዚህ ጥናት ላይ ያለዎት ተሳትፎ ከዚህ ሆስፒታል የምታገኙትን ማንኛውንም እንክብካቤ ወይም ጥቅም እንደማይጎዳ ልናረጋግጥልዎ እንወዳለን። በጥናቱ ላይ እንድትሳተፉ እየጠየቅን ያለነውም ስለ restless legs syndrome እና ተያያዥ ምክንያቶች ላይ የበለጠ ለመመራመር ስለሆነ ነው። እባክዎ የመረጃ ወረቀቱን ለማንበብ/ለማዳመጥ የሚፈልጉትን ያህል ጊዜ ይውሰዱ። በዚህ ጥናት ለመሳተፍ ፍቃደኛ ከሆናችሁ ተረድታችሁ ፈቃዳችሁን መስጠት አለባችሁ። በመቀጠልም ምላሽዎን እንዲሰጡ ይጠየቃሉ። ይህ ጥናት መጠይቅ እና የህክምና ታሪክ ግምገማን ያካትታል። ሁሉም ምላሽዎ በሚስጥር ይጠበቃል እንዲሁም ከ30-40 ደቂቃዎች ያህል ይወስዳል።

በዚህ ጥናት ለመሳተፍ ፈቃደኛ ነዎት? (አዎ ከሆነ) ፊርማዎ ________________

የመረጃ ሰብሳቢው ስም _______________________ ቀን________ ፊርማ ____________

## በአማርኛ የተዘጋጀ ቃለ-መጠይቅ

# I. ፡ የስነ-ህዝብና ማህበራዊ ጉዲዮችን በሚመለከት የተዘጋጁ ጥያቄዎች

| **ተ.ቁ** | **ጥያቄዎች** | **ምላሽ** |
| --- | --- | --- |
| 101 | ዕድሜ | ____________ ዓመት |
| 102 | የመኖሪያ ቦታ | 1. ከተማ  2. ገጠር |
| 103 | የትምህርት ደረጃ | 1. መደበኛ ትምህርት ያልተማረች  2. የመጀመሪያ ደረጃ  3. ሁለተኛ ደረጃ ትምህርት ቤት (9-12 ክፍል)  4. ኮሌጅ እና ከዚያ በላይ |
| 104 | የትዳር ሁኔታ | 1. ያላገባች  2. ባለትዳር  3. የተፋታች  4. ባል የሞተባት |
| 105 | ስራ | 1. የቤት እመቤት  2. የመንግስት ሰራተኛ  3. የግል ሰራተኛ  4. የግል ተቀጣሪ  5. ገበሬ  6. ተማሪ  7. የቀን ሰራተኞች  8. ሌላ ይጥቀሱ --- |
| 106 | አማካኝ ወርሃዊ ገቢ | __**________________** የኢትዮጵያ ብር |

# II. ከእርግዝና እና የሕክምና ታሪክ ጋር የተያያዙ ጉዳዮች

| ተ.ቁ | **ጥያቄዎች** | **ምላሽ** |
| --- | --- | --- |
| 201 | የእርግዝና ብዛት | የእርገዘና ብዛት_______  28 ሳምንታት የሆነው ______ |
| 202 | የእርግዝና ጊዜ (GA) (በሳመነታት) | ______ ሳምንታት |
| 203 | ያሁኑ እርግዝና የታቀደ ነው? | 1. አዎ  2. አይደለም |
| 204 | ፅንስ ከዚህ በፊት ጽንስ ተጨናግፎብሽ ያውቃል? | 1.አዎ  2.አይ |
| 205 | ለረጅም ግዜ የቆየ ህመም አለብዎ (HTN/DM) | 1. አዎ  2. አይደለም |
| 206 | በአሁኑ እርግዝና ወቅት ያጋጠመዎ ችግር አለ? (ለምሳሌ የደም መፍሰስ፣ የእንሽርት ውሃ መፍሰስ, የደም ግፊት መጨመር … ሌላ) | 1. አዎ (ይግለጹ) ______________  2. አይ |
| 207 | የሄሞግሎቢን (Hemoglobin) መጠን | ____________ |
| 208 | የደም ግፊት ልኬት | SBP _______  DBP ________ |
| 209 | ክብደት | _________ ኪ/ግ |
| 210 | የደም ማነስ ክኒን /አይረን ወስደሻል? | 1. አዎ  2. አይደለም  አዎ ከሆነ፣ ለስንት ወር?________ |

# III. ከአልኮል፣ ከሲጋራ እና ከጫት አጠቃቀም ጋር የተያያዙ ጥያቄዎች

| **ተ.ቁ** | **ጥያቄዎች** | **ምላሽ** |
| --- | --- | --- |
| 301 | ጫት ቅመው ያውቃሉ? | 1. አዎ 2. አይደለም  አይደለም ከሆነ ወደ ጥያቄ 303 ይዝለሉ |
| 302 | ባለፉት 30 ቀናት ውስጥ ጫት ቅመው ያውቃሉ? | 1. አዎ 2. አይደለም |
| 303 | አልኮል ጠጥተው ያውቃሉ? | 1. አዎ 2. አይደለም  አይደለም ከሆነ ወደ Q 306 ይዝለሉ |
| 304 | ባለፉት 30 ቀናት ውስጥ አልኮል ጠጥተዋል? | 1. አዎ 2. አይደለም  አይ ከሆነ ወደ Q 306 ዝለል |
| 305 | ምን አይነት አልኮል ይጠቀማሉ? ይግለጹ (ለምሳሌ፡ ቢራ፣ ጠላ፣ ጠጅ፣ አረቄ፣ ወይን፣ …) | ________፣ __________፣ ________፣ ________ |
| 306 | ካፌይን ያላቸውን መጠጦች (ለምሳሌ ሻይ፣ ቡና፣ ኮላ) ይጠጣሉ? | እባኮትን በቀን ምን ያህል ያብራሩ 1. አዎ 2. አይ  አዎ ከሆነ _______________ (ይግለጹ) |

# IV. የእንቅልፍ ንጽህና መረጃ ጠቋሚ

| **ተ.ቁ** | **የእንቅልፍ ንፅህና አጠባበቅ መረጃ ጠቋሚ** | **የጥያቄዎች ምላሽ** | | | | |
| --- | --- | --- | --- | --- | --- | --- |
| 401 | በማይመች አልጋ ላይ እተኛለሁ (ለምሳሌ ደካማ ፍራሽ ወይም ትራስ, በጣም ብዙ ወይም በቂ ያልሆነ ብርድ ልብሶች እጠቀማለሁ). | ***ሁልጊዜ*** | ***ተደጋጋሚ*** | ***አንዳንድ ጊዜ*** | ***አልፎ አልፎ*** | ***በጭራሽ*** |
| 402 | በማይመች መኝታ ክፍል ውስጥ እተኛለሁ (ለምሳሌ፡ በጣም ብርሃን የበዛብት (ያለበት)፣ በጣም የተጨናነቀ፣ በጣም ሞቃት፣ በጣም ቀዝቃዛ ወይም ጫጫታ የበዛበት )። |  |  |  |  |  |
| 403 | ከመተኛቴ በፊት አስፈላጊ ስራዎችን (ለምሳሌ: ሂሳቦችን መክፈል, የቀጣይ ውሎ እቅድ ወይም ጥናት) እሰራለሁ. |  |  |  |  |  |
| 404 | ከመተኛቴ በፊት ሊያነቃኝ የሚችል ነገር አደርጋለሁ (ለምሳሌ፡ ስልክ መደዋወል፣ የቪዲዮ ጨዋታ/game መጫወት፣ ኢንተርኔት መጠቀም ወይም ቤት ማጽዳት)። |  |  |  |  |  |
| 405 | ወደ መኝታ ከሄድኩ በኋላ ወይም ከመተኛቴ 4 ሰዓታት በፊት አልኮል, ትምባሆ ወይም ካፌይን ያላቸው ነገሮች እጠቀማለሁ |  |  |  |  |  |
| 406 | በአልጋ ላይ ስሆን አስባለሁ, አቅድዳለሁ ወይም እጨነቃለሁ. |  |  |  |  |  |
| 407 | አልጋዬን የምጠቀመው ከእንቅልፍ ውጪ ለሆኑ ነገሮችም ነው (ለምሳሌ ቴሌቪዥን ለመመልከት፣ ለማንበብ፣ ለመብላት ወይም ለማጥናት)። |  |  |  |  |  |
| 408 | ወደ መኝታ ከመሄዴ 1 ሰአት በፊት እስኪያልበኝ ድረስ የአካል ብቃት እንቅስቃሴ አደርጋለሁ። |  |  |  |  |  |
| 409 | በየቀኑ በተለያዩ ጊዜያት ከእንቅልፌ እባንናለሁ። |  |  |  |  |  |
| 410 | በየቀኑ በተለያዩ የሰአት ልዩነት እተኛለሁ. |  |  |  |  |  |
| 411 | በሳምንት ሁለት ወይም ሶስት ጊዜ መነሳት ከነበረብኝ ሰአት በላይ አልጋ ላይ እቆያለሁ. |  |  |  |  |  |
| 412 | ለሁለት ወይም ከዚያ በላይ ሰዓታት የሚቆይ የቀን እንቅልፍ እተኛለሁ። |  |  |  |  |  |
| 413 | በጭንቀት፣ በንዴት፣ በመበሳጨት ወይም በጭንቀት እየተሰማኝ ወደ መኝታ እሄዳለሁ። |  |  |  |  |  |
| 414 | አብዛሃኛውን ግዜ እንዴት መተኛት ይመርጣሉ? | A. በጀርባ  B. በግራ በኩል  C. በቀኝ ጎን  D. በደረት  E. ሌላ_____ | | | | |

# V. ደረጃውን የጠበቀ የእንቅልፍ ጥራት መለኪያ (PSQI) ጥያቄዎች

| መመሪያ፡ የሚከተሉት ጥያቄዎች ባለፈው ወር ብቻ ከተለመዱት የእንቅልፍ ልምዶችዎ ጋር ይዛመዳሉ። የእርስዎ መልሶች ባለፈው ወር ውስጥ ለአብዛኛዎቹ ቀናት እና ምሽቶች በጣም ትክክለኛውን ምላሽ ማመልከት አለባቸው።  ባለፈው ወር  501. ብዙውን ጊዜ ወደ መኝታ የሚሄዱበት ሰአት መቼ ነው? ___________________  502. በእያንዳንዱ ሌሊት ለመተኛት ምን ያህል ጊዜ (በደቂቃዎች) ፈጅቶብዎታል? _______________  503. ብዙውን ጊዜ በጠዋት የሚነሱት መቼ ነው? ___________________  504. ሀ. በምሽት ምን ያህል ትክክለኛ እንቅልፍ ያገኛሉ? (ይህ በአልጋ ላይ ከምታጠፉት የሰአታት ብዛት የተለየ ሊሆን ይችላል) _____________  ለ. በአልጋ ላይ ምን ያህል ሰዓታት ያሳልፋሉ? __________________ | | | | |
| --- | --- | --- | --- | --- |
| 505. በ*ባለፈው ወር* ምን ያህል ጊዜ ለመተኛት ተቸግረዋል ምክንያቱም እርስዎ… | በባለፈው ወር አልተከሰተም (0) | በሳምንት ከአንድ ጊዜ ያነሰ (1) | በሳምንት አንድ ወይም ሁለት ጊዜ (2) | በሳምንት ≥3 ጊዜ (3) |
| ሀ. በ30 ደቂቃ ውስጥ መተኛት ይቸገራሉ |  |  |  |  |
| ለ. በእኩለ ሌሊት ወይም በማለዳ ይነቃሉ |  |  |  |  |
| ሐ. መታጠቢያ ቤቱን ለመጠቀም ከእንቅልፍዎ ይነቃሉ |  |  |  |  |
| መ. በምቾት ለመተንፈስ ይቸገራሉ |  |  |  |  |
| ሠ. ሳል ወይም ማንኮራፋት አለዎት |  |  |  |  |
| ረ. በጣም ቅዝቃዜ ይሰማዎታል |  |  |  |  |
| ሰ. በጣም ሙቀት ይሰማዎታል |  |  |  |  |
| ሸ. መጥፎ ሕልም ያያሉ |  |  |  |  |
| ቀ. ህመም ይሰማዎታል |  |  |  |  |
| በ. በነዚህ ምክንያቶች ምን ያህል ጊዜ ለመተኛት እንደተቸገሩ ጨምሮ ሌላ ምክንያት ካለዎት እባክዎን ይግለጹ። |  |  |  |  |
| 506. ባለፈው ወር ውስጥ ለመተኛት ብለው ምን ያህል ጊዜ መድሃኒት ወስደዋል (የታዘዘ ወይም ሌላ ገዝተው)? |  |  |  |  |
| 507. ባለፈው ወር ውስጥ በጉዞ ላይ፣ ምግብ በሚመገቡበት ጊዜ ወይም በማህበራዊ እንቅስቃሴዎች ላይ ነቅተው ያለመቆየት ችግር አጋጥሞዎታል? |  |  |  |  |
| 508. ባለፈው ወር ውስጥ ነገሮችን ለማከናወን ፍላጎት የማጣት ችግር ነበር? |  |  |  |  |
| 509. ባለፈው ወር የእንቅልፍ ጥራትዎን በአጠቃላይ እንዴት ይመዝኑታል? | በጣም  ጥሩ (0) | ጥሩ (1) | መጥፎ (2) | በጣም መጥፎ (3) |
| 510. የቀን እንቅልፍ ይወስዳሉ? | 1. አዎ  2. አይ | አዎ ከሆነ፣ በአማካኝ ለምን ያህል ደቂቃ? ______ ደቂቃ | | |

# VI. ከሬስት ለስ ሌግ ሲንድሮም (Restless legs syndrome) ጋር የተያያዙ ጥያቄዎች (በIRLSSG መስፈርቶች መሰረት)

| **ተ.ቁ** | **ጥያቄዎች** | **ምላሽ** |
| --- | --- | --- |
| 601 | ከእግሮቼ ላይ በሚሰማኝ ምቾት ማጣት ወይም የማይመቹ ስሜቶች ጋር በተያያዘ እግሮቼን ማንቀሳቀስ እፈልጋለሁ። | 1. አዎ 2. አይደለም  አይደለም ከሆነ ወደ ቀጣዩ ክፍል ይለፉ |
| 602 | የእግር እረፍት አልባነት ስሜትን እና ምቾት ማጣትን ለማስታገስ ይንቀሳቀሳሉ (ለምሳሌ የእግር ጉዞ [ወክ]) ወይም እግሮችዎን ያሻሉ። | 1. አዎ 2. አይደለም |
| 603 | ምልክቶቹ በእረፍት ጊዜ የሚበረቱ ሲሆን በእንቅስቃሴ ደሞ ጊዜያዊ እፎይታ ያገኛሉ። | 1. አዎ 2. አይደለም |
| 604 | እነዚህ ምልክቶች በቀን ወይም በሌሊት ይባባሱ ነበር። | 1. አዎ 2. አይደለም |
| **ልዩ ሁኔታዎች** 1- ተሳታፊው ከዚህ ቀደም የIRLSSG መስፈርቶችን ካሟሉ እና ከዚያ በኋላ ጉልህ የሆነ የሕመም ምልክቶችን በመቀየር መድሃኒት ከወሰዱ።  **ልዩ ሁኔታዎች** 2- በሽተኛው በአንድ ወቅት የህመም ምልክቶችን በእንቅስቃሴ እፎይታ አግኝቶ የነበር ሲሆን አሁን ግን በጣም ከባድ ስለሆነ በእንደዚህ አይነት መልኩ እፎይታ ማግኘት አይችልም።  **ልዩ ሁኔታዎች** 3- ህመሙ በአንድ ወቅት በቀን ወይም በሌሊት የከፋ ነበር, አሁን ግን በጣም ከባድ ስለሆነ ምልክቶቹ ቀን እና ማታ እኩል ናቸው። | | |

# VII. የሬስት ለስ ሌግ ሲንድሮም (Restless Legs syndrome) ደረጃ አሰጣጥ ልኬት

| **ተ.ቁ** | **እረፍት የሌላቸው እግሮች ሲንድረም ደረጃ አሰጣጥ ስኬል ጥያቄዎች** | **(4) በጣም ከባድ** | **(3) ከባድ** | **(2) መጠነኛ** | **(1) መለስተኛ** | **(0) ምንም** |
| --- | --- | --- | --- | --- | --- | --- |
| 701 | በአጠቃላይ፣ በእግርዎ ወይም በእጆችዎ ላይ ያለውን የ RLS ምቾት ማጣት እንዴት ይገመግሙታል? |  |  |  |  |  |
| 702 | በአጠቃላይ፣ በእርስዎ RLS ምልክቶች ምክንያት ምን ያክል መንቀሳቀስ ያስፈልግዎታል? | በ.ያስፈልገኛል | ያስፈልገኛል | መጠነኛ | መለስተኛ | ምንም |
| 703 | በአጠቃላይ እንቅስቃሴ በማድረግ ከክንድዎ ወይም ከእግርዎ ምቾት ማጣት ምን ያህል እፎይታ ያገኛሉ? | (4)እፎይታ የለም | (3) ትንሽ እፎይታ | (2) መጠነኛ እፎይታ | (1) ሙሉ በሙሉ እፎይታ | (0) የ RLS ምልክቶች የሉም |
| 704 | በአጠቃላይ፣ ከእርስዎ የ RLS ምልክቶች የእንቅልፍዎ መረበሽ ምን ያህል አስተዋጽዎ አላቸው? | (4) በጣም ከባድ | (3) ከባድ | (2) መጠነኛ | (1) መለስተኛ | (0) ምንም |
| 705 | ከ RLS ምልክቶች የተነሳ ድካምዎ ወይም እንቅልፍ እንቅልፍ የሚለዎ ምን ያህል ከባድ ነው? | (4) በጣም ከባድ | (3) ከባድ | (2) መጠነኛ | (1) መለስተኛ | (0) ምንም |
| 706 | በአጠቃላይ፣ የእርስዎ ችግር (RLS) ምን ያህል ከባድ ነው? | (4) በጣም ከባድ | (3) ከባድ | (2) መጠነኛ | (1) መለስተኛ | (0) ምንም |
| 707 | የዚህ ችግር (RLS) ምልክቶች በሳምንት ምን ያህል ጊዜ ይከሰትብዎታል? | 6-7 ቀናት | ከ4-5 ቀናት | 2-3 ቀናት | ≤1 ቀን | ምንም የለም |
| 708 | የ RLS ምልክቶች ሲታዩ በአማካይ በቀን ምን ያህል ግዜ ይቆያሉ? | >8 ሰአታት በቀን | 3-8 ሰአታት በቀን | 1-3 ሰአት በቀን | <1ሰአት በቀን | የለም |
| 709 | በአጠቃላይ፣ የእርስዎ ምልክቶች (RLS) በእርስዎ የዕለት ተዕለት ጉዳዮች ላይ፣ ለምሳሌ አጥጋቢ ቤተሰብን፣ ቤትን፣ ማህበራዊን፣ ትምህርት ቤትን ወይም የስራ ህይወትን በመምራት ላይ ያላቸው ተጽእኖ ምን ያህል ከባድ ነው? | (4) በጣም ከባድ | (3) ከባድ | (2) መጠነኛ | (1) መለስተኛ | (0) ምንም |
| 710 | ከእነዚህ (RLS) ምልክቶችዎ የተነሳ የስሜትዎ መረበሽ ምን ያህል ከባድ ነው - ለምሳሌ መቆጣት፣ መደበር፣ ሀዘን፣ መጨነቅ ወይም መበሳጨት? | (4) በጣም ከባድ | (3) ከባድ | (2) መጠነኛ | (1) መለስተኛ | (0) ምንም |
| 711 | ባለፈው ሳምንት የነበረዎትን ምልክት እንዴት ይገመግሙታል? | 1 2 3 4 5 6 7 8  መለስተኛ መካከለኛ ከባድ በጣም ከባድ | | | | |

# VIII. የኤድንበርግ የቅድመ ወሊድ (በእርግዝና ወቅት የሚከሰት) ጭንቀት መጠንን መለኪያ ጥያቄዎች

| **ተ.ቁ** | **ባለፉት 7 ቀናት ውስጥ:** | እንደበፊቱ ነው | እንደበፊቱ አይሆንም | በጭራሽ እንደበፊቱ አይሆንም | ሙሉ ለሙሉ አልስቅም |
| --- | --- | --- | --- | --- | --- |
| 801 | መሳቅ እና የነገሮችን አስቂኝ ጎን ማየት ችያለሁ |  |  |  |  |
| 802 | ነገሮችን በደስታ አከናውናለሁ/እሰራለሁ |  |  |  |  |
| 803 | ነገሮች ሲበላሹ ራሴን ተጠያቂ አድርጌያለሁ |  |  |  |  |
| 804 | ያለ በቂ ምክንያት እጨነቃለሁ ወይም ተጨንቄአለሁ። |  |  |  |  |
| 805 | ያለ በቂ ምክንያት ፍርሃት ወይም ድንጋጤ ይሰማኛል። |  |  |  |  |
| 806 | ነገሮች ከኔ ቁጥጥር ውጭ እየወጡ ነው። |  |  |  |  |
| 807 | ደስተኛ ስላልሆንኩ ለመተኛት ተቸግሬ ነበር። |  |  |  |  |
| 808 | ሀዘን ተሰምቶኛል። |  |  |  |  |
| 809 | በጣም ደስተኛ ስላልሆንኩ እያለቀስኩ ነበር። |  |  |  |  |
| 810 | ራሴን ስለመጉዳት ሀሳብ አስቤ አውቃለሁ። |  |  |  |  |

# IX. ነብሰጡር እናቶች በእርግዝና ወቅት መስራተ ስላለባቸው የአካል ብቃት እንቅስቃሴ በተመለከተ ጥያቄ

| **ተ ቁ** | **ጥያቄዎች** | **ምላሽ** | | |
| --- | --- | --- | --- | --- |
| 901 | በሕክምና ችግር ምክንያት በእርግዝና ወቅት የአካል ብቃት እንቅስቃሴን ለመቀነስ ከጤና ባለሙያው ምክር አግኝተዋል? | 1. አዎ 2. አልሰራም | | |
| 902 | ከማርገዝዎ በፊት የአካል ብቃት እንቅስቃሴ ይሰሩ ነበር? | 1. አዎ 2. አልሰራም | | |
| 903 | በአሁኑ እርግዝና ወቅት የአካል ብቃት እንቅስቃሴ ልምምድ ያደርጋሉ? | 1. አዎ 2. አልሰራም | | |
|  |  | ሰርቸ አላዉቅም | አዎ በየቀኑ <30 ደቂቃ ወይም በየሳምንቱ <3 ቀን | አዎ በየቀኑ ≥30 ደቂቃ ወይም በየሳምንቱ ≥ 3 ቀን |
| 904 | የእግር ጉዞ(ወክ) የአካል ብቃት እንቅስቃሴ ያደርጋሉ? |  |  |  |
| 905 | በመደነስ የአካል ብቃት እንቅስቃሴ ያደርጋሉ? |  |  |  |
| 906 | ሳይክል በመንዳት የአካል ብቃት እንቅስቃሴ ያደርጋሉ? |  |  |  |
| 907 | በመተንፈስ የአካል ብቃት እንቅስቃሴ ያደርጋሉ? |  |  |  |
| 908 | የዳሌ አካል ብቃት እንቅስቃሴ ያደርጋሉ? |  |  |  |
| 909 | የጉልበትና የእግር ጣቶችን አካል ብካት እንቀስቃሴ ያደርጋሉ? |  |  |  |

**-------አመሰግናለሁ------**
