# Supplementary material for: Restless legs syndrome among third-trimester pregnant women attending ANC Clinic in public hospitals in North Wollo Zone, Ethiopia
Source: PLoS One. 2026 Oct 15;21(10):e0360405. doi: 10.1371/journal.pone.0360405 (PMC13671541; doi:10.1371/journal.pone.0360405)
Supplement: Supplementary file 2 [file pone.0360405.s003.docx]

**Point-by-point response to editor’s and reviewers’ comments**

**Journal name: PLOS ONE**

**Submission ID: PONE-D-26-08455**

**Revision: one**

**Manuscript title: Restless legs syndrome among third trimester pregnant women attending ANC Clinic in public hospitals in North Wollo Zone, Ethiopia**

First and foremost, we want to extend our heartfelt appreciation to the Academic Editor, Dr. Yuqing Li, and both reviewers for their valuable feedback and positive remarks. Below we address each point in turn. All changes have also been incorporated into the marked-up “Revised Manuscript with Track Changes” file.

|  | **Academic Editor’s Comments** | **Our responses** |
| --- | --- | --- |
| 1. | Please ensure that your manuscript meets PLOS ONE's style requirements, including those for file naming. The PLOS ONE style templates can be found at  <https://journals.plos.org/plosone/s/file?id=wjVg/PLOSOne_formatting_sample_main_body.pdf> and  <https://journals.plos.org/plosone/s/file?id=ba62/PLOSOne_formatting_sample_title_authors_affiliations.pdf> | We apologize for the inconvenience. We have reformatted the manuscript (headings, text spacing, references, tables, and file names and formats) to match the PLOS ONE style templates for main body text and title/authors/affiliations. |
| 2. | We noticed you have some minor occurrence of overlapping text with the following previous publication(s), which needs to be addressed:  <https://pmc.ncbi.nlm.nih.gov/articles/PMC8057569/>  <https://link.springer.com/article/10.1007/s11325-018-1619-5>  In your revision ensure you cite all your sources (including your own works), and quote or rephrase any duplicated text outside the methods section. Further consideration is dependent on these concerns being addressed. | We apologize for the inconvenience. After reviewing the papers, we compared our manuscript against both flagged sources sentence-by-sentence and rewrote every overlapping passage outside the Methods section in our own words in the whole section.  **Sample changes**  **Before revision**: "Restless leg syndrome (RLS) is a syndrome which manifests itself with uncomfortable sensations in the legs particularly while sleeping or resting at night, causing an urge to move the legs…"  **Revised:** "Restless leg syndrome (RLS) is a condition characterized by unpleasant feelings in the legs, especially during sleep or when at rest at night, leading to a need to shift the legs. Although patients may have difficulty describing it accurately, RLS presents as pins-and-needles, crawling, cold sensation, pressure, and warmth in the thighs, legs, and feet [1, 2]…" |
| 3. | Thank you for stating the following financial disclosure:  “The fund is to accommodate just for the data collection.”  Please state what role the funders took in the study.  If the funders had no role, please state: "The funders had no role in study design, data collection and analysis, decision to publish, or preparation of the manuscript."  If this statement is not correct you must amend it as needed.  Please include this amended Role of Funder statement in your cover letter; we will change the online submission form on your behalf. | We apologize for the inconvenience.  We confirm the funder had no role in the study. The Financial Disclosure has been amended in the cover letter to the standard PLOS wording.  **Before revision:** " Funding: Fund was received from Woldia University for this study. However, the funder has no role on the method development, conducting the study, or on preparing this manuscript.  **Revised:** "Funding: Fund was received from Woldia University to accommodate just for the data collection. However, the funders had no role in study design, data collection and analysis, decision to publish, or preparation of the manuscript." |
| 4. | We note that the grant information you provided in the ‘Funding Information’ and ‘Financial Disclosure’ sections do not match.  When you resubmit, please ensure that you provide the correct grant numbers for the awards you received for your study in the ‘Funding Information’ section. | We apologize for the inconvenience.  We have reviewed both sections. Woldia University provided a small institutional research grant to support data collection only; there is no external grant number, so we have removed the inconsistent grant reference and entered identical wording (institutional support, no grant/award number) in both the Funding Information and Financial Disclosure fields of the resubmission form.  **Before revision:** Funding Information and Financial Disclosure sections listed non-matching grant details.  **Revised:** “Fund was received from Woldia University to accommodate just for the data collection. However, the funders had no role in study design, data collection and analysis, decision to publish, or preparation of the manuscript.” |
| 5. | Thank you for stating the following in the Funding Section of your manuscript:  “Fund was received from Woldia University for this study. However, the funder has no role on the method development, conducting the study, or on preparing this manuscript.”  We note that you have provided funding information that is not currently declared in your Funding Statement. However, funding information should not appear in the Acknowledgments section or other areas of your manuscript. We will only publish funding information present in the Funding Statement section of the online submission form.  Please remove any funding-related text from the manuscript and let us know how you would like to update your Funding Statement. Currently, your Funding Statement reads as follows:  “The fund is to accommodate just for the data collection.”  Please include your amended statements within your cover letter; we will change the online submission form on your behalf.  In the online submission form you indicate that your data is not available for proprietary reasons and have provided a contact point for accessing this data. Please note that your current contact point is a co-author on this manuscript. According to our Data Policy, the contact point must not be an author on the manuscript and must be an institutional contact, ideally not an individual. Please revise your data statement to a non-author institutional point of contact, such as a data access or ethics committee, and send this to us via return email. Please also include contact information for the third party organization, and please include the full citation of where the data can be found. | We apologize for the inconvenience.  We removed the funding sentence from the manuscript body and adjust the funding statement. |
| 6. | 6. In the online submission form you indicate that your data is not available for proprietary reasons and have provided a contact point for accessing this data. Please note that your current contact point is a co-author on this manuscript. According to our Data Policy, the contact point must not be an author on the manuscript and must be an institutional contact, ideally not an individual. Please revise your data statement to a non-author institutional point of contact, such as a data access or ethics committee, and send this to us via return email. Please also include contact information for the third party organization, and please include the full citation of where the data can be found. | We apologize for the inconvenience.  The email address is [midw@wldu.edu.et](mailto:midw@wldu.edu.et) for midwifery department in Woldia University, Woldia, Ethiopia |
| 7. | Your ethics statement should only appear in the Methods section of your manuscript. If your ethics statement is written in any section besides the Methods, please delete it from any other section.  If the reviewer comments include a recommendation to cite specific previously published works, please review and evaluate these publications to determine whether they are relevant and should be cited. There is no requirement to cite these works unless the editor has indicated otherwise. | We apologize for the inconvenience.  We removed the ethics-related sentence found outside the Methods section. |
|  | **Reviewer: 1 Comments** | **Our responses** |
| 1. | 1. Clarification of RLS diagnostic criteria: The manuscript says that RLS diagnosis was made according to the “restless legs syndrome study group criteria,” but there is not enough information regarding diagnostic approach The authors should clarify: the IRLSSG diagnostic criteria were applied which criteria version was used (e.g., IRLSSG 2014 criteria) whether diagnosis was based solely on questionnaire results or confirmed by trained health care providers Symptoms of RLS can overlap with other pregnancy-related discomforts (eg, leg cramps) thus requiring a clear description of the diagnostic process. | **We apologize for the inconvenience**  The diagnosis was based on responses to a structured, interviewer-administered questionnaire covering all five criteria. We used the 2014 version. The limitations are stated at the end of the Discussion section.  **Based on your constructive comments, we refine our operational definition to be understandable for readers as follows:**  **Before revision:**  "Restless leg syndrome: a woman was considered as she has experienced restless leg syndrome when she full fill the diagnosis criteria based on restless leg syndrome study group criteria [32]."  **Revised:**  " Restless legs syndrome: A woman was classified as having restless legs syndrome if she met all five essential diagnostic criteria defined by the 2014 International Restless Legs Syndrome Study Group (IRLSSG): …  " |
| 2. | Description and validation of the questionnaire: In the manuscript it is stated a structured questionnaire was used, but there is not a clear description of components in the questionnaire. The authors should specify: whether they used a standardized RLS questionnaire or screening tool if severity was assessed by the use of Restless Legs Syndrome Rating Scale (RLSRS) how the questionnaire was translated and culturally adapted. whether the questionnaire has been previously validated. | We apologize for the inconvenience. We used expert comment and pre-test to validate the standardized questionnaire.  **Before revision:** "...using pre-tested interviewer administered questionnaire which includes: socio-demographic characteristics..., standardized restless leg syndrome related questions and medical record review checklist."  **Revised:** " …The questionnaire underwent expert validation and pretesting, and any ambiguous or unsuitable items were revised… " |
| 3. | Interpretation of prevalence findings: The 11.61% prevalence reported is lower than those from many international studies. Although the authors attribute this disparity primarily to differential genetics and healthcare systems, alternative explanations should be considered, such as:  potential differences in diagnostic methods insufficient awareness of RLS leads to underreporting differences in study population characteristics A more cautious read of these results would improve the discussion. | We appreciate your suggestion. Accordingly, we have revised the manuscript as follows:  **Before revision:** "The discrepancy in the prevalence of RLS in these studies might be related to population characteristics such as genetics and ethnic diversity [42, 43], as well as the higher prevalence of obesity in high- and middle-income countries [44]. Access to health care and well-developed prenatal care systems may influence symptom reporting..."  **Revised (expanded):** "..., 29.2% in Turkey [39], 50.3% in brazil [40], and 31.2% in New York [41].  Several factors, beyond population-level genetic and ethnic differences [42, 43], may account for this disparity.  … Access to healthcare and well-developed prenatal care systems may also influence symptom reporting, as greater clinical awareness and diagnostic familiarity with RLS in more developed health systems, such as Germany and Britain, can increase symptom elicitation independent of true biological differences [45]. Variation in study timing may be a further factor, since a single cross-sectional assessment, as used in our study, may miss transient or late-onset symptoms that longitudinal or repeated-assessment designs are more likely to capture. Given these multiple explanations, the lower prevalence observed in our study should be interpreted cautiously." |
| 4. | Limited assessment of biological risk factor: Several studies analyzing RLS throughout the gestation period examine biochemical indices like: serum ferritin. Hemoglobin folate vitamin B12The current study assessed León only iron supplementation and anemia. It is understandable that not all settings have access to laboratory parameters, especially resource-limited environments. But it needs to be explicitly stated that these data are not available and should've been discussed in the manuscript. | **We appreciated your comment and incorporated as the study limitation.**  **Before revision:** "The cross-sectional nature of the study limits the ability to establish causal relationship between the identified factors and RLS. Additionally, the reliance on self-reported data particularly on lifestyle factors may introduce recall bias which could have influenced the findings."  **Revised:** "... Despite the valuable insights provided by this study, certain limitations should be acknowledged. The cross-sectional nature of the study limits the ability to establish causal relationship between the identified factors and RLS. The reliance on self-reported data rather than clinical confirmation, other similar problems might not be entirely excluded. In addition, due to resource constraints in the setting, biochemical markers such as serum iron, ferritin, folate, and vitamin B12 were not measured. Additionally, lifestyle factors might be affected by recall bias which could have influenced the findings. " |
| 5. | Interpretation of regression findings: Results from multivariable logistic regression analysis deserve some clarification. For example: And urban residence (AOR 5.78) also showed a relatively stronger association with RLS. The discussion includes other lifestyle factors like being sedentary and getting stressed, but those explanations arenecessarily speculative. The interpretation of this finding could be strengthened by including further discussion of potential confounders (eg, socioeconomic status, reporting bias or differences in availability/access to health care). | **Before revision:** "Women residing in urban areas were 5.781 times more likely to have RLS compared to those living in rural areas... Urban environments are often associated with more sedentary lifestyles... Additionally, environmental stressors such as noise, pollution, and the fast pace of urban life..."  **Revised:** "...fast pace of urban life can contribute to higher stress levels and poor sleep quality, both of which are linked to RLS [48]. However, this association may reflect the fact that urban women often have greater access to healthcare and higher awareness of health issues, which can lead to more frequent reporting of RLS symptoms …" |
| 6. | Analysis of RLS severity: The paper reports on severity categories (mild, moderate, severe), but does not provide any further analysis. It would be interesting to see the possible associations between severity and factors such as: sleep quality depression obstetric variables If in sufficient cases such analyses are not available this limitation may be mentioned. | **Thank you for the comment. We have included it in the limitation section as follows:**  **Revised: “…**The small number of women in each RLS severity category precluded stratified analyses of RLS severity and its correlates…” |
| 7. | Misinterpretation of findings from a Turkish cohort study: The interpretation of the findings of a Turkish cohort study referenced as 39 in the discussion section seems to differ fromthe actual results In the manuscript it is written that the prevalence of RLS in Turkey was 64.4% which is wrong. The prevalence estimates: 29% as an overall prevalence of RLS during pregnancy and 64.4% for the proportion of people with RLS in third trimester, also equal to that itself, not a point estimate [2]. Suggested alternate naming convention: "Hence, the comparative analysis mentioned in manuscript can potentially resultin misinterpretation of literature." This needs to be addressed as it is misleading compared to what were previously published results. | We apologize for the inconvenience we have got your comment correct and we made correction on it.  **Before revision:** "...19% in University hospital of Aachen, Germany [23], 24.1% in King Abdulaziz Medical City in Riyadh, Saudi Arabia [38], 64.4% in Turkey [39], 50.3% in brazil [40]..."  **Revised:** "...24.1% in King Abdulaziz Medical City in Riyadh, Saudi Arabia [38], 29% in a Turkish cohort [39], 50.3% in Brazil [40]..." |
|  | **Minor Comments** | **Our response** |
| 8. | Language and grammar: The paper needs extensive editing language. Some sentences are not grammatically correct or have unclear wording. Example:“Four hundred twenty two eligible third trimester pregnant women’s were included in this study.” Suggested revision:“In this study 422 eligible third-trimester pregnant women participated.” Professional English editing is recommended. | We apologize for any inconvenience caused. We have made every effort to check and improve the language throughout the entire manuscript. |
| 9. | The manuscript alternates between: “Restless leg syndrome” “Restless legs syndrome” Use the term Restless Legs Syndrome (RLS) consistently in accordance with current medical literature. | We standardized terminology throughout the manuscript, title, abstract, and references list to "Restless Legs Syndrome (RLS)".  **Before revision:** "Background: Restless leg syndrome is uncomfortable sensations in the legs..."  **Sample Revised:** "Background: Restless Legs Syndrome (RLS) is characterized by uncomfortable sensations in the legs..." |
| 10. | Sample Size Calculation: Since there are no previous studies, a prevalence of 50% was assumed for sample size calculation. Nevertheless, there are many previous studies internationally that have addressed RLS in pregnant women. Authorsshould provide a short explanation for their choice of the prevalence in sample size calculation. | **Before revision:** "...using Epi-info software with the following assumptions: margin of error 5%, confidence level 95%, Proportion 0.5 (no similar study), and adding 10% non-response rate."  **Revised:** "...Proportion 0.5, since no prior study on RLS among pregnant women had been conducted in Ethiopia at the time of protocol design… |
| 11. | Tables and formatting: Certain tables needed better formatting, in particular: a. alignment of odds ratios and confidence interval clearer identification of reference categories | Thank you. We have reformatted the table to improve readability by separating rows with horizontal lines and shading the variable-name row. |
| 12. | Discussion section: General background information is repeated in the discussion section of the manuscript and could be shortened to focusmore clearly on the findings of our study. | Thank you. We shortened the opening paragraph of the Discussion and focused it directly on our findings.  **Before revision:** "Restless Legs Syndrome is linked to different conditions like iron deficiency, chronic illnesses, quality of life, pregnancy and pregnancy outcomes. This institution-based cross-sectional study aimed to determine the prevalence and associated factors of RLS during the third trimester of pregnancy among women attending antenatal care. The findings revealed a prevalence of 11.61%..."  **Revised:** "This study found an RLS prevalence of 11.61..." |

| 13. | **Reviewer: 2 Comments** | **Our responses** |
| --- | --- | --- |
| 14. | The topic is relevant and potentially useful because data on restless legs syndrome among pregnant women in Ethiopia are limited. However, the manuscript has substantial weaknesses in methodology reporting, statistical robustness, language quality, PLOS ONE compliance, and interpretation of findings. | We acknowledge the concerns regarding methodology reporting, statistical robustness, language quality, PLOS ONE compliance, and interpretation of findings, and have addressed each of these in the revised manuscript. |
| 15. | The manuscript states that RLS was diagnosed using the Restless Legs Syndrome Study Group criteria, but it does not fully present the diagnostic items, scoring approach, language adaptation, or validation in the local context. This is a serious issue because RLS symptoms in pregnancy may overlap with leg cramps, edema, neuropathy, anemia-related discomfort, musculoskeletal pain, and sleep disturbance. The authors should explain how these differential conditions were excluded or minimized. | We appreciated your comments.  We now explicitly state that RLS was diagnosed using the 2014 IRLSSG consensus criteria and have added the full operational definition and key diagnostic questions to the Methods section.  The questionnaire underwent expert content review, translation by language experts, and pretesting; ambiguous or unsuitable items were revised.  **Sample revision:**  “  … Restless Legs Syndrome: A woman was classified as having restless legs syndrome if she met all five essential diagnostic criteria defined by the 2014 International Restless Legs Syndrome Study Group (IRLSSG) consensus criteria: (1) an urge to move the legs; (2) the urge to move the legs and any accompanying unpleasant sensations begin or worsen during periods of rest or inactivity; (3) the sensations are partially or totally relieved by movement; (4) the sensations are worse or occur only in the evening or night than during the day; and (5) these features are not solely accounted for by another medical or behavioral condition [32].  ” |
| 16. | Evaluate and, if relevant, cite Khan et al. (2025), a systematic review/meta-analysis on IV ferric carboxymaltose for RLS. | We reviewed the suggested reference and, given its relevance to the discussion of iron's role in RLS pathophysiology, have added it to the Discussion and reference list. |
| 17. | The finding that iron supplementation is protective is plausible, but without iron-status biomarkers, it remains uncertain whether the association reflects true iron deficiency, better ANC adherence, better socioeconomic status, or other unmeasured confounding. | Thank you.  Accordingly, we have addressed this as a study limitation. Given the cross-sectional design and the resource-limited setting,  **Revised:** “… due to resource constraints in the setting, biochemical markers such as serum iron, ferritin, folate, and vitamin B12 were not measured…” |
| 18. | The model does not adequately account for several clinically relevant factors:  ferritin or iron-deficiency status hemoglobin concentration as continuous data BMI or obesity parity and gravidity in final interpretation caffeine intake chronic kidney disease family history of RLS medication use leg cramps or neuropathy socioeconomic status ANC attendance frequency  Because these are not fully controlled, the reported associations may be confounded. | We apologize for the inconvenience.  In order to make clear these limitations for future studies and readers we acknowledge that several biologically and socially plausible confounders were not available in this dataset. We have therefore added an expanded limitations statement to reflect the possibility of residual confounding.  However, our reason for some variables are:  Chronic kidney disease and Ferritin/iron status: Requires specialized lab equipment.  Hemoglobin as continuous data: Recorded categorically.  BMI/obesity: BMI is not acceptable for third trimester pregnancy.  Parity/gravidity in final model: Collected, but excluded at the p<0.25 bivariable screening step, not omitted from data collection.  Family history of RLS: Low RLS awareness in the community made reliable family-history reporting infeasible.  Medication use: Study scope limited to iron supplementation, the standard ANC intervention relevant to RLS.  Leg cramps/neuropathy: Would require specialist assessment beyond the setting and study scope.  Socioeconomic status: Income used as proxy.  ANC attendance frequency: Available but merged for report as it is not the main objective of the study. |
| 19. | The study reports that 39.1% of participants drank alcohol during pregnancy. This is a high proportion and requires careful definition. The authors should specify whether this means any alcohol use, current use, frequency, amount, traditional beverages, or binge drinking. Without dose and frequency, the variable is too crude.  There is also risk of social desirability bias, especially for alcohol use during pregnancy. | We apologize for the lack of clarity.  In this study, alcohol use during pregnancy was assessed using a single yes/no question, without capturing frequency, quantity, or beverage type.  Any reported consumption of an alcohol-containing beverage, regardless of amount, was classified as "yes,". |
| 20. | Urban residence had a very large association with RLS, but this is not well explained. Since the study was conducted in public hospitals and most participants were urban dwellers, this association may reflect sampling structure, access to care, reporting differences, lifestyle factors, or unmeasured socioeconomic variables rather than a true biological association.  The manuscript should avoid presenting urban residence as a direct risk factor without a cautious explanation. | We appreciate your comment. In light of this observation, we agree that highlighting this discrepancy may help other researchers and readers identify possible factors underlying the association with urban residence. Accordingly, we have incorporated your suggestion and added the following:  **Revised:** ". However, this association may reflect the fact that urban women often have greater access to healthcare and higher awareness of health issues, which can lead to more frequent reporting of RLS symptoms…” |

Thank you!!

We received numerous constructive comments and viewpoints, which have greatly contributed in improving our manuscript and providing valuable insights for our future work.
