# Supplementary material for: Restless legs syndrome among third-trimester pregnant women attending ANC Clinic in public hospitals in North Wollo Zone, Ethiopia
Source: PLoS One. 2026 Oct 15;21(10):e0360405. doi: 10.1371/journal.pone.0360405 (PMC13671541; doi:10.1371/journal.pone.0360405)
Supplement: Peer review history

# Peer review: Decision Letter 0

**DOI:** 10.1371/journal.pone.0360405.r001

**Authors:** Yuqing Li

5 Jul 2026

Dear Dr. Mislu,

Thank you for submitting your manuscript to PLOS ONE. After careful consideration, we feel that it has merit but does not fully meet PLOS ONE’s publication criteria as it currently stands. Therefore, we invite you to submit a revised version of the manuscript that addresses the points raised during the review process.

Please accept my apologies for the delay in reaching this decision; securing sufficient reviewers proved more time-consuming than anticipated despite a high number of invitations. I agree with the assessments provided by both reviewers and have found no conflicts between their recommendations. While the subject matter is scientifically relevant, the manuscript would benefit from a formal revision to address the reviewers' specific technical and structural points and ensure it fully meets the journal's criteria for publication.

Please submit your revised manuscript by Aug 19 2026 11:59PM. If you will need more time than this to complete your revisions, please reply to this message or contact the journal office at plosone@plos.org. When you're ready to submit your revision, log on to https://www.editorialmanager.com/pone/ and select the 'Submissions Needing Revision' folder to locate your manuscript file.

- A letter that responds to each point raised by the academic editor and reviewer(s). You should upload this letter as a separate file labeled 'Response to Reviewers'.
- A marked-up copy of your manuscript that highlights changes made to the original version. You should upload this as a separate file labeled 'Revised Manuscript with Track Changes'.
- An unmarked version of your revised paper without tracked changes. You should upload this as a separate file labeled 'Manuscript'.

If you would like to make changes to your financial disclosure, please include your updated statement in your cover letter. Guidelines for resubmitting your figure files are available below the reviewer comments at the end of this letter.

If applicable, we recommend that you deposit your laboratory protocols in protocols.io to enhance the reproducibility of your results. Protocols.io assigns your protocol its own identifier (DOI) so that it can be cited independently in the future. For instructions see: https://journals.plos.org/plosone/s/submission-guidelines#loc-laboratory-protocols. Additionally, PLOS ONE offers an option for publishing peer-reviewed Lab Protocol articles, which describe protocols hosted on protocols.io. Read more information on sharing protocols at https://plos.org/protocols?utm_medium=editorial-email&utm_source=authorletters&utm_campaign=protocols.

As the corresponding author, your ORCID iD is verified in the submission system and will appear in the published article. PLOS supports the use of ORCID, and we encourage all coauthors to register for an ORCID iD and use it as well. Please encourage your coauthors to verify their ORCID iD within the submission system before final acceptance, as unverified ORCID iDs will not appear in the published article. *Only*  the individual author can complete the verification step; PLOS staff *cannot*  verify ORCID iDs on behalf of authors.

We look forward to receiving your revised manuscript.

Kind regards,

Yuqing Li, Ph.D.

Academic Editor

PLOS One

Journal requirements:

When submitting your revision, we need you to address these additional requirements.

1. Please ensure that your manuscript meets PLOS ONE's style requirements, including those for file naming. The PLOS ONE style templates can be found at

https://journals.plos.org/plosone/s/file?id=wjVg/PLOSOne_formatting_sample_main_body.pdf and

https://journals.plos.org/plosone/s/file?id=ba62/PLOSOne_formatting_sample_title_authors_affiliations.pdf

2. We noticed you have some minor occurrence of overlapping text with the following previous publication(s), which needs to be addressed:

https://pmc.ncbi.nlm.nih.gov/articles/PMC8057569/

https://link.springer.com/article/10.1007/s11325-018-1619-5

In your revision ensure you cite all your sources (including your own works), and quote or rephrase any duplicated text outside the methods section. Further consideration is dependent on these concerns being addressed.

3. Thank you for stating the following financial disclosure:

“The fund is to accommodate just for the data collection.”

Please state what role the funders took in the study.  If the funders had no role, please state: "The funders had no role in study design, data collection and analysis, decision to publish, or preparation of the manuscript."

If this statement is not correct you must amend it as needed.

Please include this amended Role of Funder statement in your cover letter; we will change the online submission form on your behalf.

4. We note that the grant information you provided in the ‘Funding Information’ and ‘Financial Disclosure’ sections do not match.

When you resubmit, please ensure that you provide the correct grant numbers for the awards you received for your study in the ‘Funding Information’ section.

5. Thank you for stating the following in the Funding Section of your manuscript:

“Fund was received from Woldia University for this study. However, the funder has no role on the method development, conducting the study, or on preparing this manuscript.”

We note that you have provided funding information that is not currently declared in your Funding Statement. However, funding information should not appear in the Acknowledgments section or other areas of your manuscript. We will only publish funding information present in the Funding Statement section of the online submission form.

Please remove any funding-related text from the manuscript and let us know how you would like to update your Funding Statement. Currently, your Funding Statement reads as follows:

“The fund is to accommodate just for the data collection.”

Please include your amended statements within your cover letter; we will change the online submission form on your behalf.

6. In the online submission form you indicate that your data is not available for proprietary reasons and have provided a contact point for accessing this data. Please note that your current contact point is a co-author on this manuscript. According to our Data Policy, the contact point must not be an author on the manuscript and must be an institutional contact, ideally not an individual. Please revise your data statement to a non-author institutional point of contact, such as a data access or ethics committee, and send this to us via return email. Please also include contact information for the third party organization, and please include the full citation of where the data can be found.

7. Your ethics statement should only appear in the Methods section of your manuscript. If your ethics statement is written in any section besides the Methods, please delete it from any other section.

If the reviewer comments include a recommendation to cite specific previously published works, please review and evaluate these publications to determine whether they are relevant and should be cited. There is no requirement to cite these works unless the editor has indicated otherwise.

[Note: HTML markup is below. Please do not edit.]

Reviewers' comments:

Reviewer's Responses to Questions

**Comments to the Author**

1. Is the manuscript technically sound, and do the data support the conclusions?

Reviewer #1: Partly

Reviewer #2: Yes

**********

2. Has the statistical analysis been performed appropriately and rigorously?

Reviewer #1: Yes

Reviewer #2: No

**********

3. Have the authors made all data underlying the findings in their manuscript fully available?

The PLOS Data policy

Reviewer #1: Yes

Reviewer #2: Yes

**********

4. Is the manuscript presented in an intelligible fashion and written in standard English?

Reviewer #1: Yes

Reviewer #2: Yes

**********

Reviewer #1: Manuscript Title:

Restless legs syndrome among third trimester pregnant women attending ANC Clinic in public hospitals in North Wollo Zone, Ethiopia

Dear Authors,

General Comments

This is an important topic as RLS in pregnancy correlates with sleep disturbances, impaired quality of life and possiblecomplications for both mother and infant. It is true that data from low- and middle-income countries, especially from African populations are scarce; thus, this study could be valuable in broadening our knowledge of pregnancy-related RLS globally.

Nonetheless, several methodological clarifications, analytical improvements and language revisions are essential before the manuscript is potentially publishable. Although some limitations around laboratory testing may be understandable in low-resource settings, others—such as the diagnostic criteria, structure of questionnaires used, interpretation of results and comparisons to existing literature—must be re-examined.

Overall, in my opinion the manuscript has scientific merit and should be published after major revisions for bettermethodological transparency and clarity.

1. Clarification of RLS diagnostic criteria: The manuscript says that RLS diagnosis was made according to the “restless legs syndrome study group criteria,” but there is not enough information regarding diagnostic approach The authors should clarify: the IRLSSG diagnostic criteria were applied which criteria version was used (e.g., IRLSSG 2014 criteria) whether diagnosis was based solely on questionnaire results or confirmed by trained health care providers Symptoms of RLS can overlap with other pregnancy-related discomforts (eg, leg cramps) thus requiring a clear description of the diagnostic process.

2. Description and validation of the questionnaire: In the manuscript it is stated a structured questionnaire was used, but there is not a clear description of components in the questionnaire. The authors should specify: whether they used a standardized RLS questionnaire or screening tool if severity was assessed by the use of Restless Legs Syndrome Rating Scale (RLSRS) how the questionnaire was translated and culturally adapted. whether the questionnaire has been previously validated. T

3. Interpretation of prevalence findings: The 11.61% prevalence reported is lower than those from many international studies. Although the authors attribute this disparity primarily to differential genetics and healthcare systems, alternativeexplanations should be considered, such as: potential differences in diagnostic methods insufficient awareness of RLS leads to underreporting differences in study population characteristics A more cautious read of these results would improve the discussion.

4. Limited assessment of biological risk factor: Several studies analyzing RLS throughout the gestation period examine biochemical indices like: serum ferritin. Hemoglobin folate vitamin B12The current study assessed León only iron supplementation and anemia. It is understandable that not all settings have access to laboratory parameters, especially resource-limited environments.But it needs to be explicitly stated that these data are not available and should've been discussed in the manuscript.

5. Interpretation of regression findings: Results from multivariable logistic regression analysis deserve some clarification. For example: And urban residence (AOR 5.78) also showed a relatively stronger association with RLS. The discussion includes other lifestyle factors like being sedentary and getting stressed, but those explanations arenecessarily speculative. The interpretation of this finding could be strengthened by including further discussion of potential confounders (eg, socioeconomic status, reporting bias or differences in availability/access to health care).

6. Analysis of RLS severity: The paper reports on severity categories (mild, moderate, severe), but does not provide any further analysis. It would be interesting to see the possible associations between severity and factors such as: sleep quality depression obstetric variables If in sufficient cases such analyses are not available this limitation may be mentioned.

7. Misinterpretation of findings from a Turkish cohort study: The interpretation of the findings of a Turkish cohort study referenced as 39 in the discussion section seems to differ fromthe actual results In the manuscript it is written that the prevalence of RLS in Turkey was 64.4% which is wrong. The prevalence estimates: 29% as an overall prevalence of RLS during pregnancy and 64.4% for the proportion of people with RLS in third trimester, also equal to that itself, not a point estimate [2]. Suggested alternate naming convention: "Hence, the comparative analysis mentioned in manuscript can potentially resultin misinterpretation of literature." This needs to be addressed as it is misleading compared to what were previously published results.

Minor Comments

1. Language and grammar: The paper needs extensive editing language. Some sentences are not grammatically correct or have unclear wording. Example:“Four hundred twenty two eligible third trimester pregnant women’s were included in this study.” Suggested revision:“In this study 422 eligible third-trimester pregnant women participated.” Professional English editing is recommended.

2. The manuscript alternates between: “Restless leg syndrome” “Restless legs syndrome”

Use the term Restless Legs Syndrome (RLS) consistently in accordance with current medical literature.

3. Sample Size Calculation: Since there are no previous studies, a prevalence of 50% was assumed for sample size calculation. Nevertheless, there are many previous studies internationally that have addressed RLS in pregnant women. Authorsshould provide a short explanation for their choice of the prevalence in sample size calculation.

4. Tables and formatting: Certain tables needed better formatting, in particular: a. alignment of odds ratios and confidence interval clearer identification of reference categories

5. Discussion section: General background information is repeated in the discussion section of the manuscript and could be shortened to focusmore clearly on the findings of our study.

Recommendation

Major Revision: The study contributes towards an underrepresented region and offers valuable epidemiological data. Nevertheless, the manuscript needs clarification of diagnostic methodology, improvement in reporting, correction of literature interpretation and also substantial language editing to become applicable for publication.

Reviewer #2: The topic is relevant and potentially useful because data on restless legs syndrome among pregnant women in Ethiopia are limited. However, the manuscript has substantial weaknesses in methodology reporting, statistical robustness, language quality, PLOS ONE compliance, and interpretation of findings.

The manuscript states that RLS was diagnosed using the Restless Legs Syndrome Study Group criteria, but it does not fully present the diagnostic items, scoring approach, language adaptation, or validation in the local context. This is a serious issue because RLS symptoms in pregnancy may overlap with leg cramps, edema, neuropathy, anemia-related discomfort, musculoskeletal pain, and sleep disturbance. The authors should explain how these differential conditions were excluded or minimized.

Try to submit this article for better improvement.

Khan A, Kumar H, Rai KD, Saeed A, Ishtiaq J, Tanveer Alam M, Chawla S, Haque MA. Efficacy and safety of intravenous ferric carboxymaltose in the treatment of restless legs syndrome: a systematic review and meta-analysis. Frontiers in Neurology. 2025 Jan 7;15:1503342.

The finding that iron supplementation is protective is plausible, but without iron-status biomarkers, it remains uncertain whether the association reflects true iron deficiency, better ANC adherence, better socioeconomic status, or other unmeasured confounding.

The model does not adequately account for several clinically relevant factors:

ferritin or iron-deficiency status

hemoglobin concentration as continuous data

BMI or obesity

parity and gravidity in final interpretation

caffeine intake

chronic kidney disease

family history of RLS

medication use

leg cramps or neuropathy

socioeconomic status

ANC attendance frequency

Because these are not fully controlled, the reported associations may be confounded.

The study reports that 39.1% of participants drank alcohol during pregnancy. This is a high proportion and requires careful definition. The authors should specify whether this means any alcohol use, current use, frequency, amount, traditional beverages, or binge drinking. Without dose and frequency, the variable is too crude.

There is also risk of social desirability bias, especially for alcohol use during pregnancy.

Urban residence had a very large association with RLS, but this is not well explained. Since the study was conducted in public hospitals and most participants were urban dwellers, this association may reflect sampling structure, access to care, reporting differences, lifestyle factors, or unmeasured socioeconomic variables rather than a true biological association.

The manuscript should avoid presenting urban residence as a direct risk factor without a cautious explanation.

**********

what does this mean?). If published, this will include your full peer review and any attached files.

If you choose “no”, your identity will remain anonymous but your review may still be made public.

**Do you want your identity to be public for this peer review?**  For information about this choice, including consent withdrawal, please see our Privacy Policy

Reviewer #1: No

Reviewer #2: No

**********

[NOTE: If reviewer comments were submitted as an attachment file, they will be attached to this email and accessible via the submission site. Please log into your account, locate the manuscript record, and check for the action link "View Attachments". If this link does not appear, there are no attachment files.]

To ensure your figures meet our technical requirements, please review our figure guidelines: https://journals.plos.org/plosone/s/figures

You may also use PLOS’s free figure tool, NAAS, to help you prepare publication quality figures: https://journals.plos.org/plosone/s/figures#loc-tools-for-figure-preparation.

NAAS will assess whether your figures meet our technical requirements by comparing each figure against our figure specifications.

# Author comment: Author response to Decision Letter 1

**DOI:** 10.1371/journal.pone.0360405.r002

16 Sep 2026

Thank you!

Attachment Submitted filename: Response to Reviewers.docx

# Peer review: Decision Letter 1

**DOI:** 10.1371/journal.pone.0360405.r003

**Authors:** Yuqing Li

28 Sep 2026

Restless Legs Syndrome Among Third Trimester Pregnant Women Attending ANC Clinic in Public Hospitals in North Wollo Zone, Ethiopia

PONE-D-26-08455R1

Dear Dr. Mislu,

We’re pleased to inform you that your manuscript has been judged scientifically suitable for publication and will be formally accepted for publication once it meets all outstanding technical requirements.

Within one week, you’ll receive an e-mail detailing the required amendments. When these have been addressed, you’ll receive a formal acceptance letter and your manuscript will be scheduled for publication.

An invoice will be generated when your article is formally accepted. Please note, if your institution has a publishing partnership with PLOS and your article meets the relevant criteria, all or part of your publication costs will be covered. Please make sure your user information is up-to-date by logging into Editorial Manager at Editorial Manager® and clicking the ‘Update My Information' link at the top of the page. For questions related to billing, please contact billing support.

If your institution or institutions have a press office, please notify them about your upcoming paper to help maximize its impact. If they’ll be preparing press materials, please inform our press team as soon as possible -- no later than 48 hours after receiving the formal acceptance. Your manuscript will remain under strict press embargo until 2 pm Eastern Time on the date of publication. For more information, please contact onepress@plos.org.

Kind regards,

Yuqing Li, Ph.D.

Academic Editor

PLOS One

Additional Editor Comments (optional):

Reviewers' comments:

Reviewer's Responses to Questions

**Comments to the Author**

Reviewer #1: All comments have been addressed

Reviewer #2: All comments have been addressed

**********

2. Is the manuscript technically sound, and do the data support the conclusions?

Reviewer #1: Yes

Reviewer #2: No

**********

3. Has the statistical analysis been performed appropriately and rigorously?

Reviewer #1: Yes

Reviewer #2: Yes

**********

4. Have the authors made all data underlying the findings in their manuscript fully available?

The PLOS Data policy

Reviewer #1: Yes

Reviewer #2: Yes

**********

5. Is the manuscript presented in an intelligible fashion and written in standard English?

Reviewer #1: Yes

Reviewer #2: Yes

**********

Reviewer #1: I would like to thank the authors for their thorough, detailed, and constructive responses to my previous comments. All requested revisions have been successfully incorporated into the manuscript. I have no further comments or recommendations. Congratulations on a well-executed study.

Reviewer #2: (No Response)

**********

what does this mean?). If published, this will include your full peer review and any attached files.

If you choose “no”, your identity will remain anonymous but your review may still be made public.

**Do you want your identity to be public for this peer review?**  For information about this choice, including consent withdrawal, please see our Privacy Policy

Reviewer #1: No

Reviewer #2: No

**********

# Editor report: Acceptance letter

**DOI:** 10.1371/journal.pone.0360405.r004

**Authors:** Yuqing Li

PONE-D-26-08455R1

PLOS One

Dear Dr. Mislu,

I'm pleased to inform you that your manuscript has been deemed suitable for publication in PLOS One. Congratulations! Your manuscript is now being handed over to our production team.

At this stage, our production department will prepare your paper for publication. This includes ensuring the following:

* All references, tables, and figures are properly cited

* All relevant supporting information is included in the manuscript submission,

* There are no issues that prevent the paper from being properly typeset

You will receive further instructions from the production team, including instructions on how to review your proof when it is ready. Please keep in mind that we are working through a large volume of accepted articles, so please give us a few days to review your paper and let you know the next and final steps.

Lastly, if your institution or institutions have a press office, please let them know about your upcoming paper now to help maximize its impact. If they'll be preparing press materials, please inform our press team within the next 48 hours. Your manuscript will remain under strict press embargo until 2 pm Eastern Time on the date of publication. For more information, please contact onepress@plos.org.

You will receive an invoice from PLOS for your publication fee after your manuscript has reached the completed accept phase. If you receive an email requesting payment before acceptance or for any other service, this may be a phishing scheme. Learn how to identify phishing emails and protect your accounts at https://explore.plos.org/phishing.

If we can help with anything else, please email us at customercare@plos.org.

Thank you for submitting your work to PLOS One and supporting open access.

Kind regards,

PLOS One Editorial Office Staff

on behalf of

Dr. Yuqing Li

Academic Editor

PLOS One
